# The ascorbate biosynthesis pathway in plants is known, but there is a way to go with understanding control and functions

**DOI:** 10.1093/jxb/erad505

**Published:** 2024-02-01

**Authors:** Nicholas Smirnoff, Glen L Wheeler

**Affiliations:** Biosciences, Faculty of Health and Life Sciences, Exeter EX4 4QD, UK; Marine Biological Association, Plymouth PL1 2PB, UK; University of Illinois, USA

**Keywords:** GDP-mannose, iron, light response, oxidative stress, 2-oxoglutarate-dependent dioxygenases, upstream open reading frame, vitamin C, *vtc* mutants

## Abstract

Ascorbate (vitamin C) is one of the most abundant primary metabolites in plants. Its complex chemistry enables it to function as an antioxidant, as a free radical scavenger, and as a reductant for iron and copper. Ascorbate biosynthesis occurs via the mannose/l-galactose pathway in green plants, and the evidence for this pathway being the major route is reviewed. Ascorbate accumulation is leaves is responsive to light, reflecting various roles in photoprotection. GDP-l-galactose phosphorylase (GGP) is the first dedicated step in the pathway and is important in controlling ascorbate synthesis. Its expression is determined by a combination of transcription and translation. Translation is controlled by an upstream open reading frame (uORF) which blocks translation of the main GGP-coding sequence, possibly in an ascorbate-dependent manner. GGP associates with a PAS-LOV protein, inhibiting its activity, and dissociation is induced by blue light. While low ascorbate mutants are susceptible to oxidative stress, they grow nearly normally. In contrast, mutants lacking ascorbate do not grow unless rescued by supplementation. Further research should investigate possible basal functions of ascorbate in severely deficient plants involving prevention of iron overoxidation in 2-oxoglutarate-dependent dioxygenases and iron mobilization during seed development and germination.

## Teaser

Ascorbate (vitamin C) is essential for plant growth. Recent developments in understanding its functions and the control of its biosynthetic pathway via d -Man/l -Gal are reviewed.

## Introduction

**Fig. 1.**
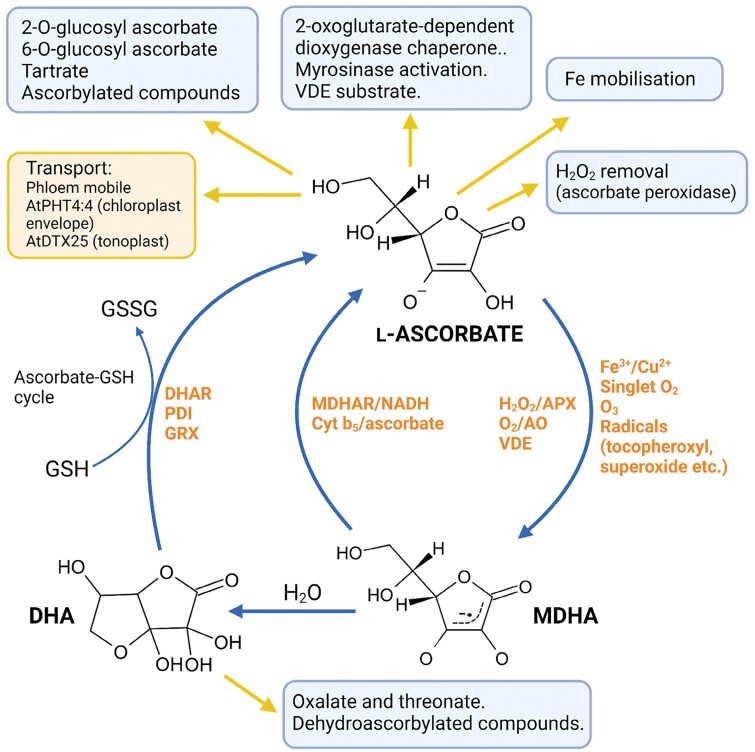
An overview of the chemistry and functions of ascorbate in plants. Ascorbic acid is predominantly present as the ascorbate anion (p*K*_a_1= 4.25). It acts as a reductant/antioxidant by reducing radicals and other reactive oxygen species by one electron transfer. H_2_O_2_ removal requires catalysis by plant-specific ascorbate peroxidases. It is unreactive with oxygen unless catalysed by ascorbate oxidase. Fe^3+^ and Cu^2+^ are readily reduced to Fe^2+^ and Cu^+^. MDHA, a resonance-stabilized radical, is the product of ascorbate oxidation. Ascorbate is an effective antioxidant because MDHA disproportionates to form DHA (most probably present as a bicyclic hemiketal form rather than the tricarbonyl structure commonly depicted) plus ascorbate. Otherwise it is reduced by MDHAR and by transmembrane reduction via cytochrome *b*_5_ which uses ascorbate as electron donor. DHA is reduced by thiols such as glutathione in the ascorbate–glutathione (Foyer–Halliwell–Asada) cycle. Ascorbate is phloem mobile and may be taken up via an unidentified plasma membrane DHA transporter, otherwise only chloroplast envelope and tonoplast ascorbate transporters have been identified. It is further metabolized to glucosides and breakdown products (in a species-dependent manner) while ascorbate and DHA can (dehydro)ascorbylate small molecules and proteins. As an antioxidant in plants, H_2_O_2_ removal using APX is the best studied function, while the physiological significance of its reactions with other radicals is less well characterized. Related to Fe, it is a protectant of the large 2-oxoglutarate-dependent dioxygen family and there is emerging evidence for a role in Fe nutrition. It is a substrate for VDE and, specifically for glucosinolate-producing species (such as Arabidopsis), it is involved in the catalytic site of myrosinases (Shikita *et al.*, 1999) which release isothiocyanates from glucosinolates following herbivore damage. Created with BioRender.com. Abbreviations: AO, ascorbate oxidase; APX, ascorbate peroxidase; DHA, dehydroascorbate; DHAR, dehydroascorbate reductase; GRX, glutaredoxin; GSH, glutathione; GSSG, oxidized glutathione; MDHA, monodehydroascorbate radical; MDHAR, monodehydroascorbate reductase; PDI, protein disulfide isomerase; VDE, violaxanthin de-epoxidase.

Ascorbate (l-ascorbic acid, vitamin C) is well known, but its functions are poorly understood (Fig. 1). As far as is known, it is restricted to eukaryotes, and it is essential for plants and mammals. Humans, other primates, bony fish, and several other groups of animals have lost the ability to synthesize ascorbate and require it in their diets. Intriguingly, this deficiency is always caused by loss of l-gulonolactone oxidase (l-GulLO), the final enzyme in the biosynthetic pathway (Duque *et al.*, 2022). Fungi synthesize d-erythroascorbate, a 5C analogue of ascorbate which has the same chemistry as ascorbate (Loewus, 1999; Baroja-Mazo *et al.*, 2005). Ascorbate is present in millimolar concentrations in plant and mammalian cells, although the latter are often deficient when grown in cell culture (Chepda *et al.*, 2001; Zhitkovich, 2020). In plants, the ascorbate concentration can be exceptionally high in fruit of some species (Fenech *et al.*, 2019), but it is generally highest in leaves and lower in roots. Typically, ascorbate concentration in lab-grown *Arabidopsis thaliana* leaves is 2–5 µmol g FW^–1^, similar in concentration to the most abundant primary metabolites sucrose, glucose, serine, and glutamate (Szecowka *et al.*, 2013). Therefore, eukaryotic cells are bathed in a relatively high ascorbate concentration which, considering that Arabidopsis mutants with ~20% of this value and cultured mammalian cells with a few percent of normal ascorbate (Zhitkovich, 2020) are still functional, suggests that ‘excess’ is maintained, possibly as an antioxidant buffer against fluctuating or unexpected conditions.

### Ascorbate chemistry and biochemical functions

The reactivity of ascorbate as a single electron (H) donor, with a relatively unreactive resonance-stabilized radical product, monodehydroascorbate (MDHA), is central to its biological function as a donor/chain-breaking antioxidant (Buettner, 1993; Buettner and Schafer, 2004; Smirnoff, 2018; Njus *et al.*, 2020). MDHA disproportionates to form dehydroascorbate (DHA; rate constant 5 × 10^5^ M^–1^ s^–1^) or is reduced to ascorbate by pyridine nucleotide-dependent MDHA reductases (MDHARs) first noted in plants by Marrè and Arrigoni (1958) (Tanaka *et al.*, 2021). DHA most probably exists as a bicyclic hemiketal structure (Fig. 1) (Njus *et al.*, 2020) which is readily reduced by thiols such as glutathione (GSH), catalysed by DHA reductases (DHARs) in the ascorbate–glutathione cycle (Foyer and Noctor, 2011). DHA comprises ~10% of the total ascorbate pool in healthy leaves, but its oxidation state varies with subcellular location and tissue type. MDHA radicals can be detected by EPR in plant tissue, particularly under oxidative stress conditions (Buettner and Jurkiewicz, 1993; Hideg *et al.*, 1997). MDHA reacts with radicals such as superoxide and tocopheroxyl radical (rate constant ~10^8^ M^–1^ s^–1^) (Njus *et al.*, 2020). Ascorbate itself reacts with and neutralizes the following biologically relevant radicals (Buettner, 1993; Buettner and Schafer, 2004; Njus, 2020): hydroxyl radical (1 × 10^10^ M^–1^ s^–1^); alkoxyl radical (1.6 × 10^9^ M^–1^ s^–1^); peroxyl radical (1 × 10^6^ M^–1^ s^–1^); thiyl radical (6 × 10^8^ M^–1^ s^–1^), superoxide (1 x 10^5^ M^-1^ s^-1^) and tocopheroxyl radical (2 × 10^5^ M^–1^ s^–1^). The biological importance of these reactions will of course depend on co-location and concentrations. It is very reactive with nitrogen dioxide radical (2 × 10^7^ M^–1^ s^–1^), poorly reactive with peroxynitrite, and unreactive with nitric oxide (Buettner and Schafer, 2004). In terms of non-radical oxidants, it has high reactivity with singlet oxygen (3 × 10^8^ M^–1^ s^–1^), forming hydrogen peroxide (H_2_O_2_) (Kramarenko *et al.*, 2006), and with ozone (4.8 × 10^7^ M^–1^ s^–1^), forming singlet oxygen (Kanofsky and Sima, 1995). Reaction with the non-radical oxidant H_2_O_2_ is very slow (2–6 M^–1^ s^–1^) (Buettner and Schafer, 2004) unless catalysed by a specialized family of ascorbate peroxidases (APXs) generally limited to photosynthetic organisms. APX acts in H_2_O_2_ removal additionally to the more widely distributed peroxiredoxins and glutathione peroxidases (Dietz, 2016; Maruta *et al.*, 2016). Ascorbate is not directly oxidized by oxygen, but in plants apoplastic Cu-containing ascorbate oxidases (AOs) catalyse oxidation to water and MDHA. The function of AO is enigmatic, but roles in cell expansion and symbiotic interactions with nitrogen-fixing bacteria and mycorrhizal fungi have been proposed (Balsetrini *et al.*, 2012; Garchery *et al.*, 2013; Chatzopoulo *et al.*, 2020).

The other key property of ascorbate is its ability to form complexes with, and reduce, higher oxidation states of transition metal ions such as Fe^3+^ and Cu^2+^. Cu^2+^ reduction is 80 times faster than that of Fe^3+^ (Buettner and Schafer, 2004). Trace concentrations of Fe and Cu oxidize ascorbate catalytically in the presence of O_2_ with production of H_2_O_2_ rather than via a redox reaction (Shen *et al.*, 2021). Ascorbate maintains 2-oxoglutarate-dependent dioxygenase (2-ODD) activity by directly reducing active site Fe(IV) and Fe(III) to Fe(II), thereby avoiding irreversible inactivation (Islam *et al.*, 2018). Famously, this is the basis of the ascorbate deficiency disease scurvy, in which loss of activity of prolyl 4-hydroxylase, an 2-ODD in the endoplasmic reticulum (ER), decreased collagen production, leading to impaired joint function and death (Arrigoni and De Tullio, 2002). 2-ODDs have many functions (Kawai *et al.*, 2014) and this aspect is discussed later. Fe^3+^ reduction by ascorbate also has potential roles in iron mobilization. The ability of ascorbate to form Fe^2+^ is also the basis of the much-discussed Fenton reaction which generates highly reactive hydroxyl radicals from Fe^2+^ and H_2_O_2_, and is proposed to be the basis of the pro-oxidant effect of ascorbate under some circumstances (Castro *et al.*, 2018). This may or may not be relevant to deleterious effects of high ascorbate on pollen function, and is discussed later.

2-*O*-Glucosyl ascorbate has been detected in leaves and fruit of diverse species (Toyoda-Ono *et al.*, 2004; Richardson *et al.*, 2020, 2021). Its concentration is generally low (<0.1 µmol g FW^–1^), but is substantial (4–11 µmol g FW^–1^) in rose hips and *Lycium barbatum* (goji berry) fruit. 2-*O*-Glycosylation (or indeed 2-*O* phosphate, sulfate, and palmitate esters as used in fish diets) stabilizes ascorbate (and erythroascorbate) against oxidation (Baroja-Mazo *et al.*, 2005; Richardson *et al.*, 2020). Identification of glucosyl transferases involved in 2-*O*-glucosyl ascorbate synthesis and determining its extent of hydrolysis could be useful in producing biofortified plants with a stable high ascorbate concentration. 6-*O*-Glucosyl ascorbate occurs in phloem of *Cucurbitaceae* where it is proposed to aid ascorbate translocation via their symplastic phloem loading mechanism (Hancock *et al.*, 2008). DHA gives rise to oxalate and threonate, with 4-*O*-oxalyl-l-threonate as an intermediate, and tartrate is a major product from ascorbate in some species (Fig. 1) (DeBolt *et al.*, 2006; Green and Fry, 2005; Truffault *et al.*, 2017).

## Ascorbate biosynthesis by plants: the backstory

**Fig. 2.**
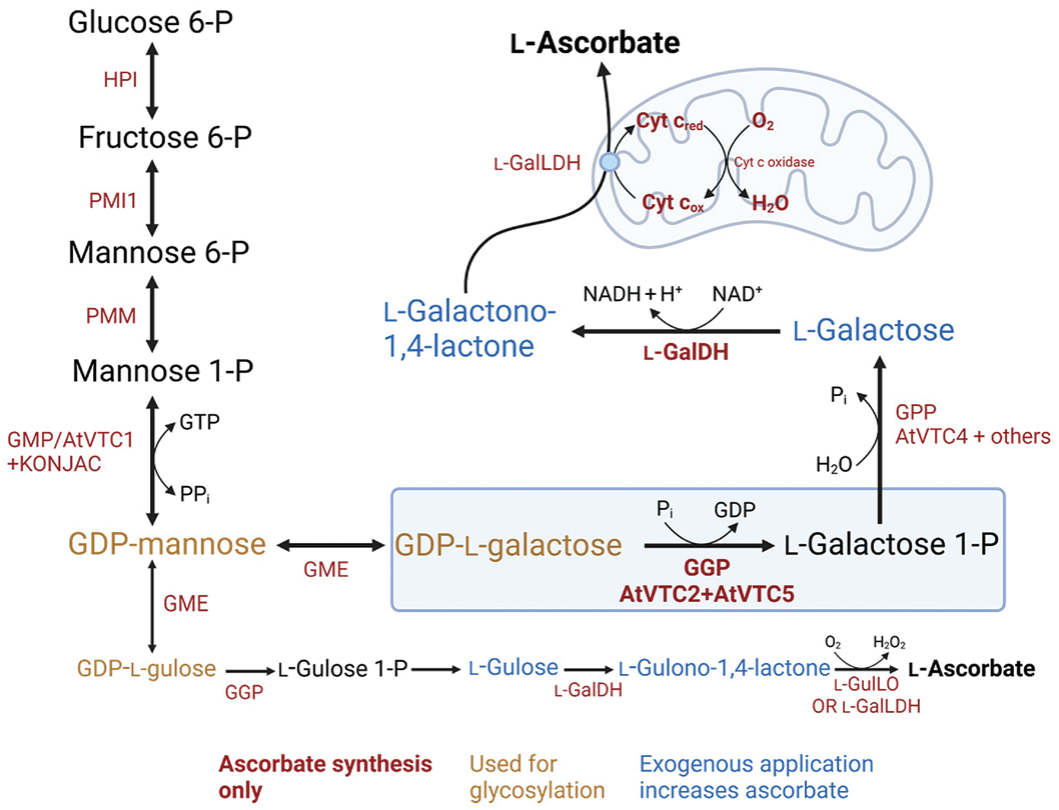
Ascorbate biosynthesis in green plants through the d-Man/l-Gal pathway. Colour coding indicates which enzymes are dedicated to ascorbate synthesis, intermediates used for glycosylation (see Fig. 3), and intermediates which are readily taken up and increase ascorbate concentration. GDP-l-galactose phosphorylase (GGP, in blue box) is the likely rate-controlling step in the pathway. Enzymes identified in ascorbate-deficient (*vtc*) Arabidopsis mutants are indicated. Created with BioRender.com. Abbreviations: l-GalDH, l-galactose dehydrogenase; l-GalLDH, l-galactonolactone dehydrogenase; GME, GDP-mannose-3',5'-epimerase; GGP, GDP-l-galactose phosphorylase; GMP, GDP-mannose pyrophosphorylase; GPP, l-galactose 1-P phosphatase; l-GulLO, l-gulonolactone oxidase; HPI, hexose phosphate isomerase; KONJAC, GDP-mannose pyrophosphorylase-like proteins activating GMP; PMI, phosphomannose isomerase; PMM, phosphomannose mutase.

Initial investigations into ascorbate biosynthesis date from the late 1950s when l-galactonolactone (l-GalL) was identified as the immediate precursor of ascorbate (Mapson and Isherwood, 1956; Isherwood and Mapson, 1962). Frank Loewus used [^14^C]glucose labelled on C1 or C6 to show that strawberry fruit produces ascorbate labelled on the same carbon atom as the glucose precursor (Loewus *et al.*, 1956; Loewus, 1999). This labelling pattern contrasts with the ‘inversion’ of the carbon skeleton in rat, suggesting that the plant and mammalian pathways differ (Smirnoff *et al.*, 2001; Smirnoff, 2011). The mammalian pathway uses UDP-d-glucuronate and l-gulonolactone as intermediates. The plant enzyme converting l-GalL to ascorbate was identified as mitochondrial l-GalL dehydrogenase (l-GalLDH) (Mapson and Breslow, 1958), again different from the mammalian equivalent l-GulLO. l-GalLDH was not characterized in more detail until nearly 40 years later (Oba *et al.*, 1995; Ostergaard *et al.*, 1997). Lycorine, an alkaloid from species in the *Amaryllidaceae*, was identified as an inhibitor of l-GalLDH and ascorbate synthesis (De Gara *et al.*, 1994), although its specificity is uncertain. Following establishment of the role of l-GalLDH and the labelling pattern in the very early work, no progress was made in identifying the pathway until Loewus and Saito proposed that d-glucosone and l-sorbosone could be precursors (Loewus *et al.*, 1990; Saito *et al.*, 1990). However, this pathway turned out to be physiologically unimportant (Pallanca and Smirnoff, 1999) in light of the proposed d-Man/l-Gal pathway, which was subsequently named the Smirnoff–Wheeler pathway by Frank Loewus (Fig. 2) (Wheeler *et al.*, 1998). The proposed pathway was based on radiolabelling and enzyme measurements. Meanwhile, Patricia Conklin and Rob Last were isolating ozone-sensitive Arabidopsis ethyl methanesulfonate (EMS) mutants. The first of these, originally named *soz1*, had decreased ascorbate content (Conklin *et al.*, 1996). The mutant was therefore renamed *vtc1*, and map-based cloning identified VTC1 as GDP-mannose pyrophosphorylase (GMP), providing the first genetic evidence for the proposed d-Man/l-Gal pathway (Conklin *et al.*, 1997, 1999). More *vtc* mutants were isolated with a high-throughput leaf squash assay detecting reduction of nitroblue tetrazolium by ascorbate (Conklin *et al.*, 2000). Six mutants from 6300 EMS-mutagenized seedlings were identified (*vtc1-2*, *vtc2-1*, *2-2*, *2-3*, *vtc3-1*, and *vtc4-1*).  VTC2 and VTC4 were eventually identified as d-Man/l-Gal pathway enzymes (Fig. 2) (Jander *et al.*, 2002; Conklin *et al.*, 2006). Map-based sequencing identified VTC3 as having a predicted N-terminal protein kinase domain and a C-terminal protein phosphatase C domain, a protein unique to green plants and red algae (Conklin *et al.*, 2013). Two EMS mutants (*vtc3-1* and *vtc3-2*) and two insertion mutants have ~35% of wild-type ascorbate and are impaired in high-light- and high-temperature-induced ascorbate accumulation. The protein phosphatase domain is predicted to be truncated in *vtc3-2* but, since all the mutants have a similar decrease in ascorbate, they are all likely to be knockouts (Conklin *et al.*, 2013). Knockout of *Physcomitrium patens VTC3* decreased ascorbate by 50%, confirming its wider role in influencing ascorbate concentration (Sodeyama *et al.*, 2021). Elucidation of the function of VTC3 in ascorbate synthesis is awaited. Because of the potential for a fully microbial-based ascorbate-manufacturing process to replace the largely chemical Reichstein process, the company Bio-Technical Resources (Wisconsin, USA) had been investigating ascorbate production in the green alga *Chlorella* and then in a heterotrophic relative *Prototheca moriformis*, filing a patent application in 1999 proposing a very similar pathway to the d-Man/l-Gal pathway (Berry *et al.*, 1999). *Prototheca moriformis* secretes ascorbate which is stable in a low pH growth medium. Mutagenesis and selection produced high ascorbate strains which had higher activity of GDP-mannose-3',5'-epimerase (GME), a d-Man/l-Gal pathway enzyme (Running *et al.*, 2002, 2003, 2004).

## Ascorbate biosynthesis by the d-mannose/l-galactose pathway

The d-Man/l-Gal pathway (Wheeler *et al.*, 1998) is summarized in Fig. 2, and is reviewed below with emphasis on newer information and gaps in knowledge. The pathway can be divided into two parts. Firstly GDP-Man and GDP-l-Gal are synthesized in a series of reactions that produce GDP-sugars for protein glycosylation and polysaccharide synthesis. Second are steps dedicated to ascorbate synthesis in which GDP-l-Gal provides l-Gal and l-GalL as the unique precursors for ascorbate. It should be noted that other pathways have been proposed, for example using d-galacturonic acid or *myo*-inositol as precursors (Broad *et al.*, 2020b). Indeed, overexpressing strawberry d-galacturonate reductase significantly increases ascorbate in Arabidopsis (Agius *et al.*, 2003) suggesting that, at least in transgenic plants, an animal-like pathway can operate.

### Enzymes involved in GDP-mannose synthesis: phosphomannose isomerase, phosphomannose mutase, and GDP-mannose pyrophosphorylase

**Fig. 3.**
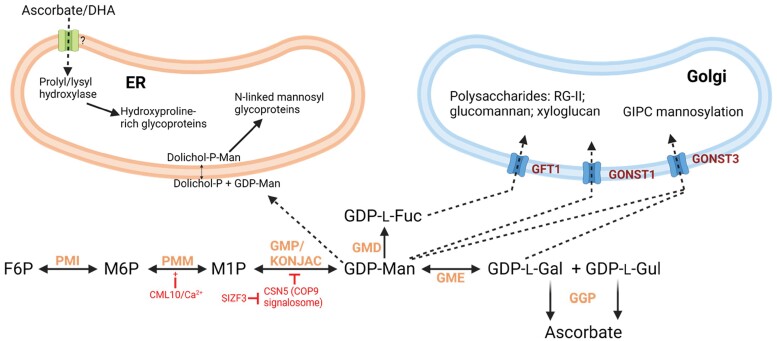
The dual role of GDP-sugars in glycosylation and ascorbate synthesis. The processing of glycoproteins destined for secretion (including structural proteins such as extensin and peptide hormones) occurs in the ER. Mannose is delivered to the ER via dolichol-P and used for protein *N*-glycoslyation. Additionally, the ER is the site of proline and lysine hydroxylation of glycoproteins by ascorbate-dependent 2-oxoglutarate-dependent dioxygenases (see Fig. 6). Glycan and glycosylinositol phosphoceramide synthesis in the Golgi uses GDP-sugars imported by various transporters. Considering the need to maintain a balance between the use of GDP-sugars for glycoproteins and polysaccharides required for cell wall production during growth and ascorbate synthesis, there is evidence that PMM is activated by interaction with a calmodulin-like protein (CML10) and GMP is subject to proteolytic breakdown by interaction with CSN5, which is antagonized by interaction of SIZF1 with CSN5. GMP is activated by two KONJAC proteins, which have a GMP-like sequence but no enzyme activity themselves. GDP-sugar availability will also be controlled by how much is used for ascorbate synthesis and, accordingly, GGP activity can act as a valve between ascorbate synthesis and glycoprotein/glycan synthesis. Created with BioRender.com. Abbreviations: DHA, dehydroascorbate; ER, endoplasmic reticulum; F6P, fructose 6-P; GDP-l-Fuc, GDP-l-fucose; GDP-l-Gal, GDP-l-galactose; GDP-l-Gul, GDP-l-gulose; GDP-Man, GDP-mannose; GIPC, glycosylinositol phosphoceramide; GMD, GDP-mannose-3,6-dehydratase=MUR1; GME, GDP-mannose-3',5'-epimerase; GMP, GDP-mannose pyrophosphorylase; GPP, l-galactose 1-P phosphatase; PMI, phosphomannose isomerase; M6P, mannose 6-P; M1P, mannose 1-P; PMM, phosphomannose mutase; RG-II, rhamnogalacturonan II.

A nexus of enzymes synthesizes a range of GDP-sugars [GDP-Man, GDP-l-Fuc, GDP-l-Gal, and GDP-l-gulose (l-Gul)] involved in protein glycosylation and glycan and sphingolipid synthesis in the ER and Golgi apparatus (Baldwin *et al.*, 2001; Sharples and Fry, 2007; Sechet *et al.*, 2018; Figueroa *et al.*, 2021; Jing *et al.*, 2021) (Fig. 3). GDP-l-Gal and GDP-l-Gul are also used for ascorbate synthesis, so control over the partitioning of GDP-Man between these functions is required, particularly in actively expanding cells. It is interesting to note that ascorbate itself is also required for maintaining the activity of ER-localized peptidyl prolyl/lysyl hydroxylases, 2-ODDs which are involved in hydroxyproline/lysine synthesis in hydroxyproline-rich glycoproteins (see section on ascorbate functions). There are suggestions of a role in oxidative cross-linking/folding of these proteins as well. Therefore, speculatively, the otherwise puzzling routing of ascorbate biosynthesis through GDP-sugars in plants could enable co-ordination of ascorbate-dependent protein hydroxylation, mannosylation, and folding in the ER.

Older observations suggested that GDP-Man is not synthesized via mannose 1/6-P because of the assumption that phosphomannose isomerase (PMI) is missing in plants. An extended VTC2 cycle involving GDP-Glc was proposed as a workaround for lack of PMI (Wolucka and Van Montagu, 2007). However, radiolabelling shows that GDP-Man is formed via PMI, rather than epimerization of GDP-Glc (Sharples and Fry, 2007), and two genes encoding PMIs were identified in Arabidopsis (Maruta *et al.*, 2008). A knockdown mutant of one (PMI1) decreases ascorbate (Maruta *et al.*, 2008), confirming its role in GDP-Man and ascorbate synthesis. Recently it was suggested that PMI1 is a moonlighting protein, interacting with the inwardly rectifying K^+^ channel KAT1, increasing stomatal aperture (Gonzalez-Garcia *et al.*, 2023).

Mannose 6-phosphate (Man 6-P) produced by PMI is converted to Man 1-P by phosphomannose mutase (PMM) (Qian *et al.*, 2007; Hoeberichts *et al.*, 2008; Badejo *et al.*, 2009). PMM is activated by Ca^2+^-dependent interaction with a calmodulin-like protein (CML10). One amiR-cml10 line has decreased ascorbate but two other amiR-cml10 lines had a greater decrease in ascorbate after H_2_O_2_ treatment (Cho *et al.*, 2016), indicating a bottleneck if ascorbate demand is increased. It would be interesting to know if Ca-dependent PMM activation has a role in controlling Man 1-P supply and avoiding a bottleneck in GDP-Man production if demand increases. However, a kinetic model of the pathway suggests that PMI and PMM are not strong control points (Fenech *et al.*, 2021).

GDP-mannose pyrophosphorylase (GMP) catalyses the reversible formation of GDP-Man from Man 1-P (Fig. 3). Its role in ascorbate synthesis was first demonstrated by identification of VTC1 (=CYT1, SOZ1) as a GMP in Arabidopsis (Conklin *et al.*, 1999) and by antisense suppression in potato (Keller *et al.*, 1999). *vtc1-1* and *vtc1-2* have a Pro22Ser substitution, ~30% ascorbate, and ~50% residual enzyme activity (Conklin *et al.*, 1999). An Arabidopsis mutant with a truncated VTC1 protein (*cyt1*) is embryo lethal (Nickle and Meinke, 1998; Lukowitz *et al.*, 2001). There are two other genes in Arabidopsis (At3g55590 and At4g30570) with very high sequence similarity to VTC1. Inspection of transcriptome data shows that they have very low expression compared with *VTC1*, explaining the embryo lethality of VTC1 knockout (Lukowitz *et al.*, 2001). Nothing is known about the function of these homologues. Rice has three GMPs which may contribute differentially to root and leaf activity (Qin *et al.*, 2016), and OsVTC1-1 RNAi lines have altered cell wall mannose composition as well as lower ascorbate (Lamanchai *et al.*, 2022). Additionally, there are two proteins in Arabidopsis, KONJAC1 and 2 (KJC1/KJC2), with ~31% sequence similarity to VTC1. They have two extra amino acids in the conserved pyrophosphorylase domain, and the recombinant His-tagged proteins lack NDP-sugar pyrophosphorylase activity. *kjc1* and *kjc2* mutants have decreased ascorbate, and *kjc1* has decreased content of GDP-Man as well as cell wall mannose. The double mutant has severe growth defects and does not flower (Sawake *et al.*, 2015). Interestingly KJCs interact with VTC1 in pull-down assays and increase the GMP activity of VTC1 by 100% (KJC1) and 50% (KJC2). A crystal structure of VTC1 shows that it dimerizes and dodecamerizes, and will provide a useful basis for future understanding of how KJC binds and activates VTC1 (Zhang *et al.*, 2022).

Because GDP-Man is needed for protein *N*-glycosylation and synthesis of Man- and l-Gal-containing polysaccharides and also Man, Fuc, and cellulose in cell walls (Lukowitz *et al.*, 2001), the *vtc1* mutants are affected in numerous functions additionally to ascorbate synthesis. Several GDP-sugar transporters are located in the Golgi membrane (Baldwin *et al.*, 2001; Rautengarten *et al.*, 2016; Sechet *et al.*, 2018; Jing *et al.*, 2021). Perturbation of glycosphingolipid synthesis in the Golgi apparatus in a mutant of the GDP-sugar transporter GONST1 increases salicylic acid (SA) and activates a constitutive hypersensitive cell death response reminiscent of other lesion mimic mutants (Mortimer *et al.*, 2013). Therefore GDP-Man shortage in *vtc1* mutants clearly impacts processes other than ascorbate biosynthesis. *vtc1* is hypersensitive to ammonia (Qin *et al.*, 2008; Barth *et al.*, 2010; Zhang *et al.*, 2021). This is not the case for other *vtc* mutants, suggesting that it is specific to GDP-Man, and the defect is suggested to be related to altered protein mannosylation or NO (Qin *et al.*, 2008; Barth *et al.*, 2010). Further investigation suggests that *vtc1* has more NO and ammonium-induced *S*-nitrosoglutathione reductase (GSNOR). Since GSNOR is required for ammonium tolerance, overexpression in *vtc1* improved its ammonium tolerance (Zhang *et al.*, 2021). Therefore, the use of *vtc1* alone to infer functions of ascorbate is unreliable. Given that VTC1 is at a crossroads where hexoses are shared between ascorbate and cell wall/glycosylation, it is interesting that several controls over its activity have been uncovered. Arabidopsis VTC1 interacts with CSN5B, which is part of the COP9 signalosome complex. This enables proteolysis via the 26S proteasome in the dark. Seedlings of a *csn5b* mutant had somewhat higher ascorbate when grown under a day–night cycle and a markedly decreased loss of ascorbate in extended dark (48 h). These results indicate that VTC1 activity is controlled via proteolysis (Wang *et al.*, 2013; Ma *et al.*, 2022). Further investigation showed that a D27E mutation in CSN5B stops interaction with VTC1 and increases ascorbate when expressed in Arabidopsis (Li *et al.*, 2016). A C2H2 zinc finger protein (SIZF3) from tomato increases ascorbate when overexpressed in tomato and Arabidopsis, and appears to work by binding CSN5B, preventing it from interacting with VTC1 (based on yeast two-hybrid and transient expression studies) (Y. Li *et al.*, 2018). Overall, although VTC1 is not dedicated to ascorbate synthesis, the control of its expression, protein turnover, and activity revealed by these studies suggest the importance of balancing GDP-Man production for use in polysaccharides required for growth, protein mannosylation, and ascorbate synthesis.

### GDP-mannose 3',5'-epimerase

GDP-mannose 3',5'-epimerase (GME) is present in all green plants/algae and beyond (Beerens *et al.*, 2021), and is encoded by one gene in Arabidopsis and either one or two genes in many other species (Watanabe *et al.*, 2006; Mounet-Gilbert *et al.*, 2016; Qi *et al.*, 2017; Tao *et al.*, 2018). GME is a cytosolic enzyme (Qi *et al.*, 2017; Fenech *et al.*, 2021) and reversibly converts GDP-Man into a mixture of GDP-l-Gal and a smaller amount of GDP-l-Gul (Wolucka *et al.*, 2001; Wolucka and Van Montagu, 2003, 2007; Major *et al.*, 2005; Watanabe *et al.*, 2006). Its crystal structure and mechanism have been determined (Major *et al.*, 2005). The products of the enzyme are multifunctional: GDP-l-Gal is transported into the Golgi via the GDP-sugar transporter GONST3/GGLT1 (Sechet *et al.*, 2018) and used for synthesis of the cell wall polysaccharide rhamnogalacturonan II (RG-II) (Gilbert *et al.*, 2009; Voxeur *et al.*, 2011; Mounet-Gilbert *et al.*, 2016). In tomato, which has two GME isoforms, RNAi knockdown showed that both contribute to ascorbate synthesis. In contrast, RNAi suppression of one of these (SlGME1) impacted pollen development and pollination, resulting in small fruit with few seeds (Mounet-Gilbert *et al.*, 2016). Borate supplementation of the tomato GME RNAi lines restores growth. Similarly, in Arabidopsis, two T-DNA insertion mutants in its single GME gene have 20–50% of wild-type ascorbate. They show reduced fertility due to impaired pollen development and germination, and this phenotype is not reversed by ascorbate or borate supplementation. Vegetative growth is greatly decreased in the mutants and is rescued by borate but not ascorbate supplementation (Qi *et al.*, 2017). GME mutants in tomato have fewer l-Gal residues in RG-II, resulting in less cross-linking in the wall and therefore impaired growth. Borate mediates cross-linking, explaining its ability to rescue growth (O’Neill *et al.*, 2004; Voxeur *et al.*, 2011). Squash (*Cucurbita pepo*) root tips have a very large decrease in growth rate and ascorbate concentration when transferred to boron-free medium (Lukaszewski and Blevins, 1996), suggesting an additional direct link between boron and ascorbate; however, this result has no simple explanation.

### GDP-l-galactose phosphorylase

**Fig. 4.**
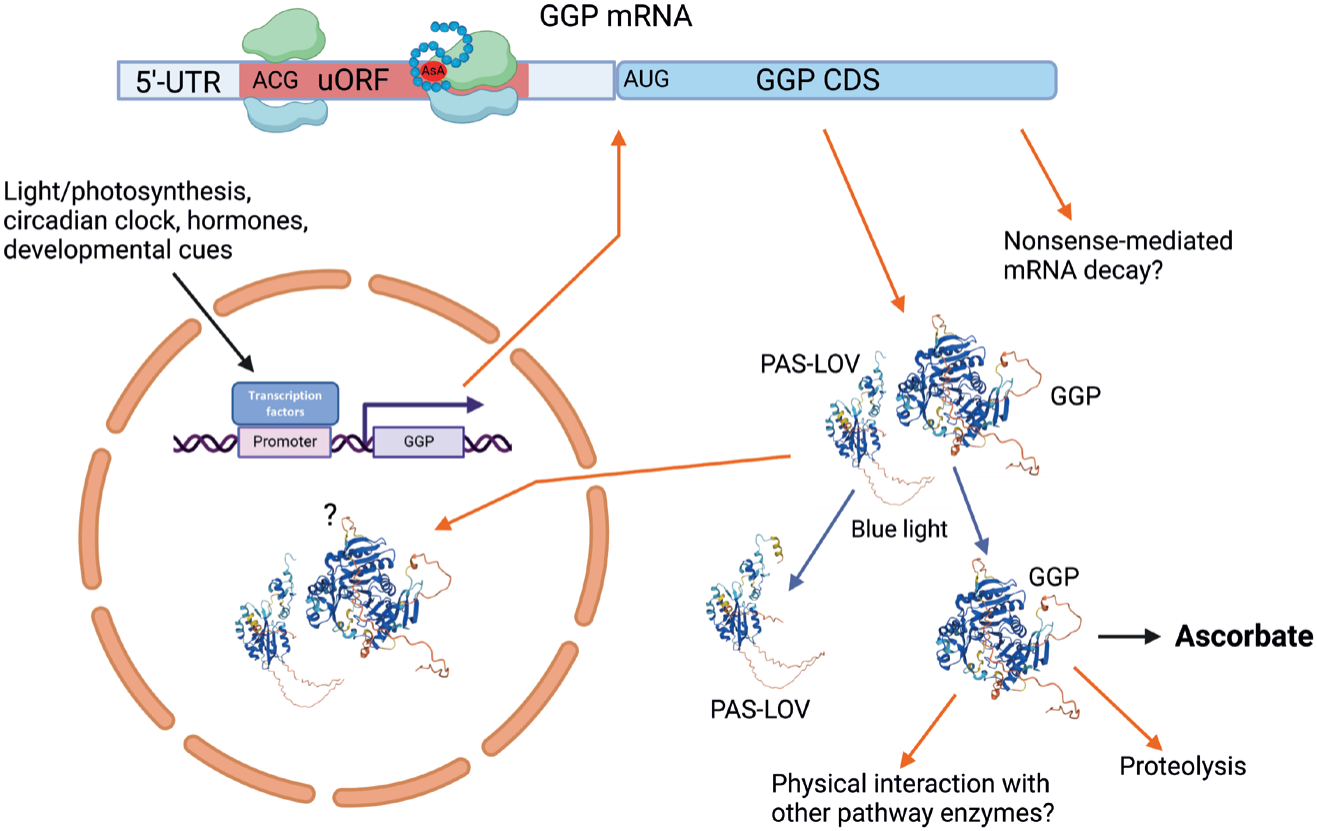
GDP-l-galactose phosphorylase (GGP) is generally the most rate-controlling step in ascorbate synthesis by the d-Man/l-Gal pathway and its activity is controlled at transcriptional, translational, and post-translational levels. Expression of *GGP* genes is responsive to many environmental factors (particularly light), and an increasing number of transcription factors are being identified. The 5'-UTR of the transcript has an upstream ORF (uORF) with a non-canonical ACG initiation codon. Ribosomes binding to the uORF stall and decrease translation of the GGP protein. It is proposed that the uORF produces a peptide which, in the presence of ascorbate, stalls the ribosomes, providing a mechanism for feedback control of ascorbate synthesis. The role of the peptide and whether it interacts with ascorbate are currently unknown. Transcripts harbouring uORFs are susceptible to degradation by nonsense-mediated decay, so GGP transcript levels may be influenced by this process as well as the rate of transcription. Once translated, GGP interacts with a PAS-LOV protein (PLP), with similarity to blue light-sensing phototropins. The complex is enzymatically inactive and is dissociated by blue light. This mechanism provides an additional light control over GGP. GGP and PLP are also located in the nucleus, but the significance of this is unknown. GGP and other d-Man/l-Gal pathway enzymes physically interact, again with unknown consequences. GGP also has predicted phosphorylation sites whose function has not been assessed. Created with BioRender.com. The GGP (AtVTC2) and PAS-LOV (AtPLP) protein structures were predicted by AlphaFold (https://alphafold.ebi.ac.uk/).

Production of l-Gal-1 from GDP-[^14^C]Man was detected in pea seedling extracts, and this activity was stimulated by phosphate (Wheeler, 2000). Subsequently GDP-l-Gal phosphorylase (GGP) was purified. Based on this work and the identification of *VTC2* by map-based cloning (Jander *et al.*, 2002), Dowdle *et al.* (2007) showed that GGP is encoded by two paralogous genes in Arabidopsis (*VTC2/AtGGP1* and *VTC5/AtGGP2*). The enzyme was also characterized by Linster *et al.* (2007, 2008) and Laing *et al.* (2007). Green plants generally have two (sometimes more) paralogues, although chlorophytes have a single copy (Tao *et al.*, 2020). In Arabidopsis, *VTC2/AtGGP1* has an ~10 times higher transcript level than *VTC5/*A*tGGP2*. The corresponding knockout mutants have 20% and 80% of normal leaf ascorbate, respectively (Dowdle *et al.*, 2007), indicating that the paralogues somehow produce a fixed proportion of ascorbate and cannot compensate each other. Double *vtc2 vtc5* mutants can only grow beyond seed germination if supplemented by ascorbate, l-Gal, or l-GalL (Dowdle *et al.*, 2007; Lim *et al.*, 2016), indicating that GGP is specific for ascorbate synthesis in Arabidopsis and that other pathways are not quantitatively significant in germinating seedlings. There is strong evidence that GGP is often the rate-controlling enzyme in ascorbate biosynthesis (Fig. 4), and this is discussed in more detail in the section on pathway control.

GGP has a dual cytosolic and nuclear localization (Muller-Moulé, 2008; Fenech *et al.*, 2021). It is a member of the histidine triad (HIT) family of nucleoside monophosphate hydrolases/transferases but lacks one of the conserved histidines, thus favouring reaction with phosphate rather than hydrolysis (Linster *et al.*, 2007). GGP is equally active with GDP-l-Gal and GDP-Glc, with *K*_m_ values of 4–10 µM (Linster *et al.*, 2008). It also shows guanylyltransferase activity, transferring GMP from GDP-l-Gal to hexose 1-P to form GDP-hexose and l-galactose 1-phosphate (l-Gal 1-P) (Laing *et al.*, 2007). However, this activity is very small (<10%) compared with phosphorolysis (Linster *et al.*, 2008). GGP is reversible, but GDP-l-Gal formation is likely to be negligible *in vivo*: the *K*_m_ for l-Gal 1-P is 45 mM and the *k*_cat_/*K*_m_ is 10^4^ times smaller than for the forward direction (Linster *et al.*, 2008). The *K*_m_ for phosphate is 1–2 mM (Linster *et al.*, 2008), while cytosolic concentrations are in the range 1–10 mM and do not drop until severe starvation (Versaw and Garcia, 2017). It is therefore conceivable that phosphate limits GGP activity in some cases. Arabidopsis mutants of an abscisic acid (ABA)-inducible PTP-like nucleotidase, which can hydrolyse GDP, (d)GMP, and (d)IMP, have ~30% of wild-type ascorbate in seedlings, apparently rescued by phosphate supplementation (Zhang *et al.*, 2020). It was proposed that the nucleotidase activity increases phosphate availability. While this conclusion is somewhat plausible, *VTC1*, *VTC2*, and *VTC5* transcripts are lower in the mutant, perhaps suggesting that the nucleotidase mutation has a more pervasive effect on gene expression. There is one other enzyme with similar catalytic properties to GGP (At5g18200 in Arabidopsis) with a two histidine HIT domain but otherwise low sequence similarity. It catalyses phosphorolysis of ADP-Glc and, although its crystal structure has been determined, its function is unknown (McCoy *et al.*, 2006).

GGP is generally green plant specific (but not obviously present in rhodophytes), although similar proteins can be found in metazoans and scattered protists (Wheeler *et al.*, 2015). The *Caenorhabditis elegans* enzyme is active against GDP-Glc and has a much lower *k*_cat_ with GDP-Man and GDP-l-Gal. Knockout in *C. elegans* causes GDP-Glc accumulation and it is proposed to function in recycling GDP-Glc produced as a side reaction of GDP-mannose pyrophosphorylase (Adler *et al.*, 2011). Synthesis or function of GDP-Glc in plants is not mentioned in a recent review on nucleotide sugars (Figueroa *et al.*, 2021), and radiolabelling suggests that it is not a major nucleotide sugar (Sharples and Fry, 2007). However, it is possible that GGP will remove GDP-Glc (formed accidentally or otherwise), while preserving GDP, as in *C. elegans*. Measurement of GDP-Glc in GGP mutants would be informative and one can speculate that an enzyme more widely used for nucleotide-sugar salvage has been co-opted for ascorbate biosynthesis in plants.

### l-Galactose 1-P phosphatase

l-Gal 1-P is hydrolysed to l-Gal by l-Gal 1-P phosphatase (GPP). An enzyme with this activity was purified and characterized from kiwifruit and Arabidopsis (Laing *et al.*, 2004a). LC-MS analysis of a tryptic digest identified a *myo*-inositol 1-P (IMP)-type protein. The recombinant enzymes strongly prefers l-Gal 1-P, *myo*-inositol 1-P, and *myo*-inositol 3-P over other phosphates, with a *K*_m_ of ~0.02–0.10 mM (Laing *et al.*, 2004a; Torabinejad *et al.*, 2009; Saxena *et al.*, 2013; Nourbakhsh *et al.*, 2014). The Arabidopsis enzyme encoded by *VTC4* was shown to be the same as the enzyme purified by Laing *et al.* (2004a) (Conklin *et al.*, 2006). Identification of further *vtc4* T-DNA knockout lines showed that plants retained ~30% of normal ascorbate, which suggests that other phosphatases can hydrolyse l-Gal 1-P (Conklin *et al.*, 2006; Torabinejad *et al.*, 2009; Saxena *et al.*, 2013). In Arabidopsis, there are two other IMP-like (IMPL) enzymes also sensitive to Ca^2+^ and Li^+^ inhibition. One of these (IMPL1) has a preference for IMP and d-Gal 1-P, while l-Gal 1-P supports 7% of activity (Nourbakhsh *et al.*, 2014). The ascorbate concentration of an *impl1* mutant is not known but, given its predicted chloroplast location and low activity with l-Gal 1-P, it is unlikely to contribute significantly to ascorbate synthesis.

Knockout of *VTC4* (*vtc4-2*, *4-3*, and *4-4*) decreases ascorbate to ~30% of wild-type levels but also decreases *myo*-inositol to ~70% of wild-type concentrations. Seed germination is delayed, and germination and seedling root growth are more sensitive to cold (Torabinejad *et al.*, 2009). Sensitivity is reversed by complementation with a chickpea VTC4 (Saxena *et al.*, 2013). Because *myo*-inositol and related compounds are involved in signalling/stress responses (Chaouch and Noctor, 2010), interpretation of the function of ascorbate using *vtc4* mutants is not advised.

### l-Galactose dehydrogenase

The key that enabled the ascorbate biosynthesis pathway to be unlocked was the discovery that l-Gal fed to plant tissues causes a rapid and large increase in ascorbate (Wheeler *et al.*, 1998) to the same extent as observed with l-GalL some decades earlier (Isherwood *et al.*, 1954). Therefore, an enzyme able to oxidize l-Gal to l-GalL was postulated and detected as NAD-dependent l-Gal dehydrogenase (l-GalDH) activity (Wheeler *et al.*, 1998). Purification from pea and N-terminal sequencing identified a potential Arabidopsis gene. The recombinant enzyme had l-GalLDH activity with a relatively high affinity for l-Gal (0.1–0.4 mM) and lower affinity for l-Gul (4 mM) and l-Fuc (56 mM) (Gatzek *et al.*, 2002). Similar properties were found for spinach and kiwifruit enzymes, with no activity supported by other sugars (Laing *et al.*, 2004b; Mieda *et al.*, 2004). Reversible competitive inhibition by ascorbate (*K*_i_ 0.1 mM) (Mieda *et al.*, 2004) or slow inactivation of kiwifruit enzyme, protected by pre-addition of NAD (Laing *et al.*, 2004b), have been reported, but no significant inhibition occurs if the pH is controlled (Vargas *et al.*, 2022). l-GalDH is predicted to be cytosolic, confirmed by transient expression of a green fluorescent protein (GFP) fusion (Fenech *et al.*, 2021). The crystal structure has been determined and is typical of the aldehyde-keto reductase (AKR) family (Vargas *et al.*, 2022). The lack of a normally conserved active site arginine explains the preference for NAD over NADP. Many of the AKR family catalyse reduction (favoured by the reduced state of NADPH), while l-GalDH should operate in the oxidative direction for ascorbate synthesis, taking advantage of the predominance of NAD^+^ over NADH in the cytosol. Overexpression of l-GalDH in tobacco has no effect on ascorbate, while antisense suppression in Arabidopsis (to 30–50% of wild-type activity) only causes a small decrease in ascorbate, evident only in high-light conditions, which causes increased ascorbate in wild-type plants (Gatzek *et al.*, 2002). These results indicate that l-GalDH exerts little control over ascorbate synthesis (Fenech *et al.*, 2021). Complete knockout of l-GalDH in a T-DNA mutant results in growth arrest after germination of homozygous seedlings, and growth is fully restored by ascorbate supplementation (Fenech *et al.*, 2021). This shows that only one enzyme in Arabidopsis can catalyse the reaction and that it is likely that its only function is ascorbate biosynthesis.

### l-Galactono-1,4-lactone dehydrogenase: ascorbate synthesis and mitochondrial Complex 1 assembly

**Fig. 5.**
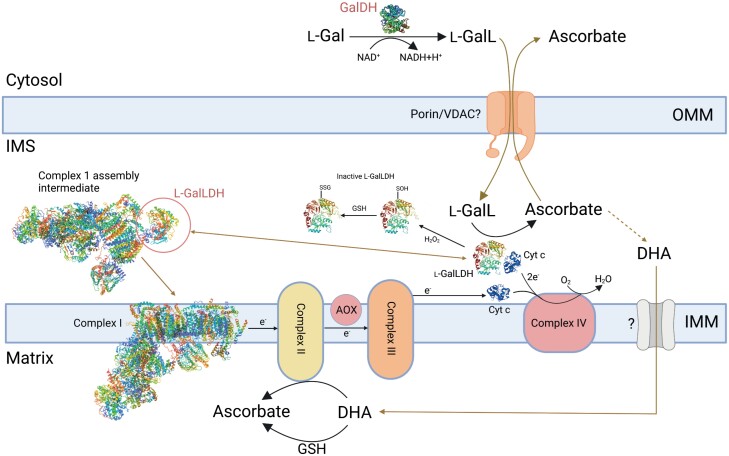
The final step of ascorbate synthesis is localized in mitochondria. l-GalL produced in the cytosol by l-GalDH enters the intermembrane space, presumably via carriers on the outer mitochondrial membrane (e.g. porins/VDACs). l-GalL is oxidized to produce ascorbate by l-GalLDH which transfers electrons to loosely associated Cyt *c* via an FAD cofactor. The reduced Cyt *c* transfers electrons to oxygen with production of water in Complex IV. Ascorbate leaves the mitochondrion, possibly thorough porin. Ascorbate enters the mitochondrial matrix as DHA. Mitochondria take up DHA in preference to ascorbate, but the IMM transporter is not identified. DHA is reduced in the matrix by GSH or by Complex II. GalLDH is oxidized by H_2_O_2_*in vitro* on a specific cysteine, resulting in an inactive sulfenic acid form which can be glutathionylated. GalLDH activity increases in the light, but it is not known if oxidation has a role *in vivo*. Remarkably, l-GalDH is also an essential component of Complex 1 assembly and is not present in the mature complex. The mitochondrial electron transport chain is not shown in detail. The Complex 1 cryo-EM and l-GalDH crystal structure are from the Protein Data Bank (https://www.rcsb.org) (accession nos 7A24, 7A23, and 7SMI). The l-GalLDH structure was predicted by AlphaFold (https://alphafold.ebi.ac.uk/). Created with BioRender.com. Abbreviations: AOX, alternative oxidase; DHA, dehydroascorbate; GSH, glutathione; IMM, inner mitochondrial membrane; IMS, intermembrane space; l-Gal(DH), l-galactose (dehydrogenase); l-GalL(DH), l-galactonolactone (dehydrogenase); OMM, outer mitochondrial membrane; VDAC, voltage-dependent anion channel.

l-Galactono-1,4-lactone dehydrogenase (l-GalLDH) from mitochondria was the first plant ascorbate biosynthesis enzyme to be identified (Mapson and Breslow, 1958). Further work confirmed its location in the inner mitochondrial membrane associated with respiratory Complex 1 (Fig. 5) (Siendones *et al.*, 1999; Bartoli *et al.*, 2000, 2003; Millar *et al.*, 2003). Isolated mitochondria convert l-GalL to ascorbate, although excess interferes with electron transport and phosphorylation along with increasing reactive oxygen species (ROS) production (Mazorra Morales *et al.*, 2022). A knockout mutant reveals that l-GalDH is also required for correct assembly of Complex 1 (Pineau *et al.*, 2008; Schertl *et al.*, 2012; Schimmeyer *et al.*, 2016), and a recent cryo-EM structure shows its location in a Complex 1 assembly intermediate (Soufari *et al.*, 2020). l-GalLDH mutants are therefore compromised in Complex 1 function in addition to ascorbate deficiency.

l-GalLDH is a member of the vanillyl-alcohol oxidase (VAO) family, as are the animal (l-GulLO) and fungal (d-arabinonolactone oxidase) enzymes. However, the key differences of the plant enzyme are that it is a dehydrogenase rather than a H_2_O_2_-producing oxidase, the FAD cofactor is not covalently linked to the enzyme (a conserved His missing), and it is mitochondrial (Leferink *et al.*, 2008). l-GalLDH has a strong preference for l-GalL (*K*_m_=0.17 mM, *k*_cat_=134 s^–1^) over l-GulL (*K*_m_=13.1 mM, *k*_cat_=4.0 s^–1^) and other aldonolactones (Mapson and Breslow, 1958; Leferink *et al.*, 2008). Transfer of two electrons from l-GalL reduces the associated FAD to a hydroquinone which then transfers electrons in two steps (with a semiquinone radical intermediate) to cytochrome *c* (Cyt *c*) (Leferink *et al.*, 2008). A transient low affinity complex between Cyt *c* and l-GalLDH enables electron transfer from reduced FAD (Hervas *et al.*, 2013). Leferink *et al.* (2008) have provided important information on Arabidopsis l-GalLDH function by using site-directed mutants. They show that mutation of a conserved valine near the flavin-binding site increases its reactivity with oxygen, explaining why the plant enzymes are dehydrogenases in contrast to other VAO enzymes (Leferink *et al.*, 2009a). A conserved Glu386 is required for substrate binding and preference for l-GalL over l-GulL, while a neighbouring Arg388 stabilizes the negative charge of the reduced flavin (Leferink *et al.*, 2009b).

The location of l-GalLDH in mitochondria and the ability of Cyt *c* to act as the electron acceptor invite the possibility that ascorbate synthesis and respiration interact with each other. Considering a typical Arabidopsis leaf respiration rate of 12 µmol O_2_ g^–1^ h^–1^ (O’Leary *et al.*, 2017) and measured ascorbate turnover rate (0.1 µmol g^–1^ h^–1^ in a leaf with 5 µmol g^–1^ at steady state; see section on control), ascorbate synthesis is unlikely to exceed a few percent (~1%) of the respiration rate, suggesting that it is not quantitatively significant and unlikely to impact respiratory flux *in vivo*. However, addition of l-GalL to intact tissue or isolated mitochondria increases l-GalLDH activity greatly and, even in this case, the leaf respiration rate did not differ between the wild type and *vtc2* (10–20% of wild-type ascorbate) with or without l-GalL feeding (Senn *et al.*, 2016). Experiments with isolated mitochondria show an interaction with the mitochondrial electron transport chain (mETC) (Siendones *et al.*, 1999; Bartoli *et al.*, 2000; Millar *et al.*, 2003; Matos *et al.*, 2022; Mazorra Morales *et al.*, 2022). Once ascorbate is synthesized in the intermembrane space, it must move to the cytoplasm and the mitochondrial matrix (Fig. 5). Presumably, l-GalL and ascorbate could move via porins/voltage-dependent anion channels (VDACs) on the outer mitochondrial membrane. Involvement of a specific transporter on the inner mitochondrial membrane has not been demonstrated but, similarly to mammals, mitochondria isolated from BY-2 cells take up DHA and not ascorbate into the matrix (Szarka *et al.*, 2004). While enzymes of the ascorbate–GSH cycle occur in mitochondria (Jimenez *et al.*, 1997), there is also evidence that DHA is reduced via electron transport from Complex II (Szarka *et al.*, 2007).

It is striking that the increase in ascorbate caused by l-Gal(L) feeding is light stimulated and inhibited by photosynthetic electron transport inhibitors (Smirnoff, 2000; Bartoli *et al.*, 2006; Yabuta *et al.*, 2008). Also, Arabidopsis acclimated to increased light over 2 weeks had greater l-GalLDH and Cyt *c* oxidase activity (Bartoli *et al.*, 2006). Furthermore, l-GalLDH activity could be influenced by the redox state of Cyt *c* and mETC activity. However, in intact mitochondria, provision of Complex 1 substrates stimulates ascorbate synthesis from l-GalL and is reversed (inhibited) by rotenone (Millar *et al.*, 2003), suggesting that it is required for maximum ascorbate synthesis rather than being competitive. However, antimycin A slightly stimulates ascorbate production (Bartoli *et al.*, 2000). These observations point to a complex picture of the control of l-GalLDH activity. Since the light response is quick, a possible explanation could be a post-translational modification. l-GalLDH is inactivated by H_2_O_2_*in vitro*, and removal of a critical cysteine near the active site by site-directed mutagenesis prevents inactivation. This Cys is sequentially oxidized to sulfenic, sulfinic, and sulfonic forms. The sulfenic form can be *S*-glutathionylated, preventing further oxidation but also switching off enzyme activity (Leferink *et al.*, 2009c). This work was carried out *in vitro*, so the physiological significance is not established. However, Leferink *et al.* (2009c) point out that this could explain light-dependent l-GalL oxidation. We need to establish if the enzyme is more reduced in high light and oxidized/glutathionylated in the dark. The extent to which this step exerts control on ascorbate synthesis is also unclear. A kinetic model, not specifically including light control at this step, suggests that most control resides with GGP (Fenech *et al.*, 2021), but both the mechanism and role in control should be investigated further.

Some GDP-l-Gul is formed by GME and could be a source of ascorbate (Fig. 2). GGP, l-GalDH, and GalLDH can use l-Gul-containing substrates, although less effectively than l-Gal (Gatzek *et al.*, 2002; Linster *et al.*, 2007; Leferink *et al.*, 2008). Another possibility is oxidation of l-GulL by plant l-GulLO-like enzymes. Arabidopsis has seven of these, and some could be functional (Maruta *et al.*, 2010; Aboobucker *et al.*, 2017; Murgia *et al.*, 2023). Overexpression of Arabidopsis l-GulLO2, 3, and 5 (as well as rat l-GulLO) in tobacco BY-2 cells increases the rate of l-GulL conversion to ascorbate (Maruta *et al.*, 2010), although transient expression of GulLO5 in *Nicotiana benthamiana* had a very small effect on l-GulL conversion to ascorbate (Aboobucker *et al.*, 2017).

## Diversity and evolution of ascorbate biosynthesis

The d-Man/l-Gal pathway for ascorbate biosynthesis found in plants is entirely distinct from the biosynthetic pathway found in animals, with no shared enzymes. The two pathways result in a different orientation of the carbon chain in the ascorbate molecule. In animals, the carbon chain from hexose sugars is inverted (carbon 1 of glucose becomes carbon 6 of ascorbate), whereas in plants the carbon chain is not inverted (carbon 1 of glucose is retained as carbon 1 of ascorbate). A survey of eukaryote genomes suggests that the core d-Man/l-Gal pathway via GGP and l-GalLDH is found only in land plants and green algae (*Viridiplantae*) (Wheeler *et al.*, 2015). Outside of the vascular plants, experimental evidence to support this pathway has been demonstrated in bryophytes (*Marchantia* and *Physcomitrium*) (Sodeyama *et al.*, 2021; Ishida *et al.*, 2023), chlorophytes (*Chlamydomonas*) (Urzica *et al.*, 2012a; Vidal-Meireles *et al.*, 2017), and trebouxiophytes (*Chlorella* and *Prototheca*) (Renstrom *et al.*, 1983; Running *et al.*, 2003).

Functional studies of ascorbate biosynthesis in these lineages have demonstrated that GGP plays a conserved role as the key controlling step in the d-Man/l-Gal pathway. However, these studies have highlighted important differences in the control mechanisms, particularly in response to light. The moss *Physcomitrium patens* contains three paralogues of *GGP* (*VTC2-1*, *VTC2-2*, and *VTC2-3*), two of which are strongly transcriptionally up-regulated by light (Sodeyama *et al.*, 2021). The light-dependent induction of both genes is strongly suppressed by the addition of the photosynthetic electron transport inhibitor DCMU [3-(3,4-dichlorophenyl)-1,1-dimethylurea]. Knockout of either gene resulted in a substantial reduction of cellular ascorbate (46% or 17% of the wild type for *vtc2-1* and *vtc2-2*, respectively), indicating that GGP makes a major contribution to ascorbate biosynthesis in moss. In contrast, the liverwort *Marchantia polymorpha* contains a single *VTC2* gene whose expression is not increased by light or oxidative stress. However, *MpVTC2* was essential for growth, as knockout *Marchantia* plants could only be maintained through supplementation with l-Gal (Ishida *et al.*, 2023).

Control of ascorbate biosynthesis via GGP also differs substantially in the green alga *C. reinhardtii*. Although high light increases cellular ascorbate, transcript levels of *CrVTC2* are not elevated in cells acclimated to high light (Vidal-Meireles *et al.*, 2017). *CrVTC2* transcripts are strongly increased by oxidative stress, suggesting that redox status rather than light may be the major factor controlling ascorbate biosynthesis in *Chlamydomonas* (Urzica *et al.*, 2012a; Vidal-Meireles *et al.*, 2017). Whilst inhibition of photosynthetic electron transport with DCMU decreases *VTC2* expression in vascular plants and *Physcomitrium*, it results in an increase in *CrVTC2* transcripts in *Chlamydomonas*. The increased expression may be triggered by the production of singlet oxygen by DCMU and demonstrates that photosynthetic electron transport is not required for elevated *CrVTC2* expression. The d-Man/l-Gal pathway is the major contributor to ascorbate biosynthesis in *Chlamydomonas*, with *CrVTC2* artificial miRNA (amiRNA) knockdown lines exhibiting just 10% of the ascorbate content of wild-type cells (Vidal-Meireles *et al.*, 2017). The observed regulatory differences are likely to be due to differences in the cellular concentrations and roles of ascorbate between these lineages. Bryophytes and green algae have a much lower ascorbate content than vascular plants (Gest *et al.*, 2013). Moreover, *Chlamydomonas* does not exhibit a strong requirement for ascorbate in the xanthophyll cycle, as severe ascorbate deficiency in *Chlamydomonas CrVTC2* knockout lines does not impair energy-dependent quenching (qE) and violaxanthin de-epoxidation (Vidal-Meireles *et al.*, 2019).

Red algae possess most of the biosynthetic enzymes of the d-Man/l-Gal pathway, including GME, l-GalDH, and l-GalLDH, but crucially lack GGP. Labelling studies demonstrated that the carbon chain of glucose is not inverted during ascorbate biosynthesis in *Galdieria sulphuraria*, indicating that red algae probably operate a modified d-Man/l-Gal pathway in which the conversion of GDP-l-Gal to l-Gal is catalysed by alternative enzymes that currently remain unidentified (Wheeler *et al.*, 2015). Red algae have the capacity to produce GDP-l-Gal as an ascorbate precursor because it is a precursor for l-Gal and 3,6-anhydro-l-Gal residues in agar polysaccharides (Su and Hassid, 1962; Falshaw *et al.*, 2023). Given that GGP plays a critical role in controlling ascorbate biosynthesis in land plants and green algae, the future identification of these enzymes in red algae will provide important insight into the evolution of these processes. A possible candidate is the ADP-glucose phosphorylase identified in Arabidopsis (McCoy *et al.*, 2006), since red algae have similar proteins.

Outside the *Archaeplastida* (red and green algae), there is little evidence to support the presence of the d-Man/l-Gal pathway in any other photosynthetic eukaryote. The *Archaeplastida* obtained their chloroplasts from a primary endosymbiotic event with a cyanobacterium, whereas plastids in other photosynthetic eukaryotes derive from a secondary endosymbiosis with a red or green alga. Remarkably, almost all photosynthetic eukaryotes with secondary plastids possess l-GalLDH, whereas non-photosynthetic eukaryotes that are capable of ascorbate biosynthesis possess l-GulLO. Experimental evidence from *Euglena*, diatoms, and chrysophytes indicates that the production of l-GalL in these photosynthetic protists requires inversion of the carbon chain (C1 of glucose becomes C6 of ascorbate) (Shigeoka *et al.*, 1979; Helsper *et al.*, 1982; Grun and Loewus, 1984). Photosynthetic eukaryotes with secondary plastids therefore appear to possess a hybrid of the animal and plant pathways, combining inversion of the carbon chain with l-GalLDH as the terminal enzyme, using d-galacturonate and l-GalL as intermediates. The distribution of these pathways supports an evolutionary scheme in which the animal pathway represents the ancestral pathway of ascorbate biosynthesis. Red and green algae subsequently evolved an entirely novel pathway in which l-GalLDH replaced l-GulLO as the terminal enzyme, and l-Gal was utilized as the precursor for l-GalL (Wheeler *et al.*, 2015). When other eukaryotes subsequently acquired photosynthesis via endosymbiosis with a green or red alga, it appears that only l-GalLDH was recruited by the host organism, leading to the formation of the hybrid pathway. In support of this hypothesis, two basally derived lineages in the red and green algae (the extremophile red alga *Galdieria sulphuraria* and the streptophyte alga, *Chlorokybus atmophyticus*) possess l-GulLO rather than l-GalLDH, but otherwise possess all other aspects of the d-Man/l-Gal pathway (Wheeler *et al.*, 2015). This suggests that the d-Man/l-Gal pathway operates with l-GulLO as the terminal oxidase in these lineages, so that the recruitment of l-Gal as an intermediate in the pathway may have pre-dated the replacement of l-GulLO with l-GalLDH. Interestingly, both *G. sulphuraria* and *C. atmophyticus* usually occupy low-light environments, suggesting that selective pressure to replace l-GulLO with l-GalLDH in the red/green algal lineages may be linked to a role in photoprotection (Wheeler *et al.*, 2015).

The strong selective pressure to replace l-GulLO with l-GalLDH in photosynthetic eukaryotes is also demonstrated by nearly all lineages that acquired their plastids via secondary endosymbiosis. Replacement of l-GulLO with l-GalLDH in these lineages would have uncoupled ascorbate production from H_2_O_2_ production, and may therefore have allowed photosynthetic eukaryotes to accumulate much larger quantities of ascorbate, enabling roles as an antioxidant and in photoprotection. Further elucidation of the nature of ascorbate biosynthesis and its cellular roles in diverse photosynthetic protists is required to test these evolutionary hypotheses.

## Ascorbate transport

Subcellular fractionation and immunocytochemical detection using ascorbate-specific antibodies indicate that ascorbate occurs in chloroplasts, mitochondria, peroxisomes, and vacuoles in millimolar concentrations (Foyer and Noctor, 2011; Koffler *et al.*, 2014). DHA predominates in the apoplast because of ascorbate oxidase (AO) activity (Pignocchi *et al.*, 2003) coupled with the limited capacity for reduction via the thiol system as shown by full oxidation of the roGFP–Orp1 H_2_O_2_ biosensor targeted to the apoplast (Arnaud *et al.*, 2023). The plasma membrane has a carrier-mediated ascorbate–DHA exchanger: DHA is taken up from the apoplast in exchange for ascorbate (Horemans *et al.*, 2000), and in *Betula pendula* the *K*_m_ for DHA uptake is 12.8 mM (Kollist *et al.*, 2001). Critically, there has been no progress in molecular identification of any plasma membrane DHA/ascorbate transporters since the review by Horemans *et al.* (2000). Interestingly, H_2_O_2_ specifically induces ascorbate efflux from cultured cells, possibly via this exchanger (Parsons and Fry, 2010). Chloroplasts take up ascorbate in a carrier-dependent manner (Foyer and Lelandais, 1996) using a Δψ-dependent transporter (AtPHT4;4) from the PHOSPHATE TRANSPORTER 4 (PHT4) family. The *pht4:4* knockout mutant has decreased leaf ascorbate content in high light. Chloroplast ascorbate was not measured in the mutant, but a decreased capacity for non-photochemical quenching (NPQ), which is dependent on the thylakoid lumen enzyme violaxanthin de-epoxidase (VDE) and which uses ascorbate as a substrate, suggests that chloroplast ascorbate is affected (Miyaji *et al.*, 2015). AtPHT4:1 could be a thylakoid membrane ascorbate transporter (Miyaji *et al.*, 2015). Mitochondrial ascorbate transport is reviewed in the discussion of l-GalLDH (Fig. 5), but specific transport proteins have not been identified. Very recently, a tonoplast-localized ascorbate transporter AtDTX25 in the multidrug and toxic compound extrusion (MATE) family was identified (Hoang *et al.*, 2021a). It is active in ascorbate transport when expressed in yeast and *Xenopus* oocytes, and its role in iron mobilization is described later. In summary, there is still much to be learnt about plant ascorbate and DHA transporters, which are clearly different from those in mammals (Smirnoff, 2018).

Unlike animals, where ascorbate biosynthesis capacity is confined to liver or kidney, it is likely that ascorbate biosynthesis is cell autonomous but with differences in concentration between tissues. As a broad generalization, ascorbate concentration is higher in photosynthetic tissue than in roots. Reproductive tissues and meristems may have relatively high concentration, while fruits vary from low to exceptionally high (camu-camu, Kakadu plum, and kiwi fruit reaching 60–200 µmol g^–1^ FW). Ascorbate seems to move from source to sink tissues via the phloem. [^14^C]Ascorbate applied to source leaves of tomato, *Medicago sativa*, and Arabidopsis followed by autoradiography of whole plants shows label in sink tissues, including reproductive parts and root tips (Franceschi and Tarlyn, 2002; Badejo *et al.*, 2011). Ascorbate occurs in potato and Arabidopsis phloem sap collected from aphids (Franceschi and Tarlyn, 2002; Tedone *et al.*, 2004) and may even be synthesized *in situ* (Hancock *et al.*, 2004). Furthermore, increasing source leaf ascorbate by feeding l-Gal(L) also increases ascorbate in sink tissues and supports its direct translocation (Franceschi and Tarlyn, 2002; Tedone *et al.*, 2004). Nevertheless, it seems that translocation generally does not provide a significant amount of fruit ascorbate (Hancock *et al.*, 2007; Li *et al.*, 2010; Badejo *et al.*, 2011).

## Control of ascorbate concentration

The difference in ascorbate concentration between tissues, and its response to environmental (light, temperature, and mineral nutrient supply) or hormonal cues, implies that ascorbate status is sensed and then adjusted to the appropriate concentration. The final concentration is obviously dependent on the balance between synthesis and breakdown.

Leaf ascorbate concentration remains relatively constant over day/night cycles, but decreases substantially in extended dark (Smirnoff and Pallanca, 1996; Dowdle *et al.*, 2007; Conklin *et al.*, 2013; Truffault *et al.*, 2017) which, in barley leaves, can be partially reversed by adding Glc or Suc (Smirnoff and Pallanca, 1996), suggesting that degradation is associated with the carbon starvation response (Pal *et al.*, 2013). Turnover rate in leaves is ~2% of the pool size per hour as measured by breakdown of [^14^C]ascorbate in Arabidopsis leaves in the light (Conklin *et al.*, 1997), potato leaves in light and dark (Imai *et al.*, 1999), and tomato leaves over 24 h in the dark (Truffault *et al.*, 2017). This means that an equivalent of about half the steady-state ascorbate concentration is replaced every 24 h in leaves. In contrast, turnover in embryos from germinating pea seeds is faster at 13% of pool size per hour (Pallanca and Smirnoff, 2000). Possibly this higher rate reflects the use of ascorbate in hydroxyproline-rich glycoprotein synthesis during rapid growth (see section on ascorbate functions). Breakdown is concomitant with oxalate and threonate accumulation in tomato leaves (Truffault *et al.*, 2017). Oxalate and threonate are well-established products of DHA breakdown via 4-*O*-oxalyl-l-threonate (Green and Fry, 2005), but to date enzymes that catalyse these reactions are unknown, so DHA availability may be the main factor. DHA concentration (typically ~10% of the total ascorbate pool) is influenced by the rate of ascorbate oxidation and the capacity to reduce it via the ascorbate–GSH cycle. A comprehensive review of many overexpression experiments shows that increasing the recycling capacity by overexpressing DHAR tends to increase ascorbate by up to 2-fold, while MDHAR overexpression has less effect (Broad *et al.*, 2020b).

Feedback inhibition is a common mechanism to prevent excessive accumulation of end products, and there is evidence that ascorbate synthesis is subject to feedback inhibition. The rate of [^14^C]ascorbate synthesis from [^14^C]Glc decreases as the ascorbate pool size increases (Pallanca and Smirnoff, 2000; Wolucka and Van Montagu, 2003). Although inhibition of some of the d-Man/l-Gal pathway enzymes *in vitro* has been reported, the effects might be artefacts due to pH or pro-oxidant effects of ascorbate, as discussed in the Introduction (Fenech *et al.*, 2021). On the contrary, the emerging evidence suggests that flux through the d-Man/l-Gal pathway is largely controlled by GGP in a complex manner (Fig. 4). There have been many attempts to increase ascorbate by overexpressing the d-Man/l-Gal pathway enzymes. As expected, results vary between species and tissues, but a comprehensive review of these experiments indicates that GGP overexpression usually increases ascorbate while the other enzymes have a small or variable effect (Bulley *et al.*, 2009; Yoshimura *et al.*, 2014; Broad *et al.*, 2020b; Fenech *et al.*, 2021). Possibly, when overexpression of enzymes involved in GDP-Man metabolism increases ascorbate, it could be because competition with mannosylation reactions is large, for example in actively growing tissue. Added to this evidence, quantitative trait locus (QTL) analysis shows that allelic variation of MdGGP1 and MdGGP3 is associated with apple fruit ascorbate concentration (Mellidou *et al.*, 2012). The metabolic engineering and genetic evidence is replicated by a kinetic model of the d-Man/l-Gal pathway which predicts that GGP is the only significant controlling step in the pathway (Fenech *et al.*, 2021). For this prediction to hold, it is necessary to include feedback inhibition of GGP by ascorbate, while reported feedback of the other enzymes has no effect. Inspection of Arabidopsis transcriptome data shows that *GGP1* and *GGP2* (*VTC2*/*VTC5*) transcript levels are more responsive to environmental factors affecting ascorbate, such as light/darkness, than the other d-Man/l-Gal pathway enzymes, and the GGPs follow a circadian rhythm under continuous light. The increased GGP transcript level in high light is reflected by increased GGP enzyme activity in Arabidopsis (Dowdle *et al.*, 2007). The discovery that GGP mRNA has a conserved upstream ORF (uORF) in its 5'-untranslated region (UTR) has been pivotal in understanding the control of ascorbate synthesis by opening up the possibility that the uORF controls translation in a ascorbate-dependent manner (Fig. 4) (Laing *et al.*, 2015). uORFs control translation of the main ORF of a significant proportion of genes by causing ribosome stalling or by activating nonsense-mediated decay (NMD) of the mRNA. In some cases, the uORF encodes a peptide which aids stalling. Examples of uORF-mediated control of metabolism include feedback repression of translation of transcription factors or biosynthetic enzymes, for example in polyamine and sucrose synthesis (Kurihara *et al.*, 2009, 2018; van der Horst *et al.*, 2020). The key points are that the GGP uORF has a non-canonical initiation codon (ACG) and is predicted to encode a peptide. Use of transiently expressed constructs in *N. benthamiana* containing the 5'-UTR/uORF fused to luciferase (LUC) reporters showed that translation is increased if the uORF is deleted or mutated, and is greatly decreased if the ACG is converted to the normal AUG initiation codon. Furthermore, increasing the ascorbate content of *N. benthamiana* by co-infiltrating with 35S::GGP lacking the uORF represses translation of the LUC reporter. Laing *et al.* (2015) proposed that translation of GGP mRNA is repressed because the interaction of the uORF-encoded peptide with ascorbate causes ribosomes to stall on the uORF and thereby blocks their progression to the AUG start of the GGP-coding sequence. Currently, details of this mechanism need clarification. The peptide has not been detected but could remain bound to the ribosomes to cause stalling. There is currently no direct evidence that ascorbate itself, or a proxy of ascorbate status, is involved in enhancing stalling. Further work confirms the importance of the GGP uORF in controlling ascorbate synthesis. A high ascorbate tomato from an EMS mutagenesis screen was mapped to the predicted uORF of SlGGP1. CRISPR/Cas9 [clustered regularly interspaced palindromic repeats (CRISPR)/CRISPR-associated protein 9] gene editing to disrupt the SlGGP1 uORF produced tomato fruit with greatly increased ascorbate by up to 5-fold (Deslous *et al.*, 2021). Using gene editing to increase ascorbate in this manner is clearly an effective metabolic engineering strategy, and its general applicability is confirmed by increased ascorbate in lettuce, Arabidopsis, and tomato following uORF mutation (T. Li *et al.*, 2018; Zhang *et al.*, 2018). However, SlGGP1 uORF edited lines of tomato with very high ascorbate have developmental defects, particularly parthenocarpy possibly caused by impaired anther development and poor pollen germination (Deslous *et al.*, 2021). The lesson is that control of ascorbate concentration at the ‘correct’ level is important for plant function, and the uORF is a key player. Further metabolic engineering strategies using the uORF will need be tuned appropriately. Another consideration in relation to the uORF is that ribosome stalling could result in targeting of the GGP mRNA by NMD. Interestingly, the *AtGGP1/VTC2* transcript level is increased in RNA helicase, UP frameshift mutants (*upf1-1* and *upf3-1I*) in the NMD process (Kurihara *et al.*, 2009). If this is the case, then the measured GGP transcript levels could be determined by a combination of transcription and destruction by NMD.

Considering the importance of ascorbate in photosynthesis and photoprotection (Toth, 2023), it is not surprising that ascorbate concentration in leaves is increased by high light in many species. For example, in Arabidopsis, adjustment to light intensity takes 5 d, with the final concentration saturating at a photosynthetic photon flux density (PPFD) of ~500 µmol m^–2^ s^–1^ (Page *et al.*, 2012). Expression of VTC2 and VTC5 promoter/5'-UTR::luciferase in Arabidopsis revealed that luminescence increased after transfer to high light and showed a circadian rhythm. Overall, the VTC5 construct had lower luciferase activity than VTC2, reflecting their transcript levels (Gao *et al.*, 2011). Since this construct contains the uORF, it is reporting the transcriptional and translational control of VTC2/5, so it will be necessary to disentangle these in future work given that transcript levels are higher in high light. Ascorbate and GGP expression are also light responsive in *Physcomitrium* (Sodeyama *et al.*, 2021) but not in *M. polymorpha* (Ishida *et al.*, 2023). Critically, the signals involved in high-light-induced GGP transcription/translation need to be identified. Inhibition of photosynthetic electron transport by DCMU decreases light-induced ascorbate accumulation and expression of GGP isoforms in Arabidopsis and *Physcomitrium* (Sodeyama *et al.*, 2021; Yabuta *et al.*, 2007). This observation suggests involvement of a photosynthesis-sourced signal. H_2_O_2_ could be ruled out because its production is blocked by DCMU (Exposito-Rodriguez *et al.*, 2017). The *VTC2* promoter has predicted light response elements (Gao *et al.*, 2011). The green alga *Chlamydomonas* responds differently: ascorbate accumulation and GGP expression are increased by DCMU, rose Bengal (a singlet oxygen generator), and H_2_O_2_ (Vidal-Meireles *et al.*, 2017, 2019). *Chlamydomonas* also accumulates ascorbate in high light, but it is suggested that this response is associated with increased H_2_O_2_ generated in the light (Vidal-Meireles *et al.*, 2017). A recent notable development in relation to the control of GGP and ascorbate synthesis by light is the role of a physical interaction between GGP and a PAS-LOV (PLP) protein. The PAS-LOV protein contains an FMN chromophore and is a putative blue light receptor which interacts with Arabidopsis GGP1 and 2 in a yeast two-hybrid assay. The interaction is weakened by blue light (Ogura *et al.*, 2008). This interaction was found to have functional significance following discovery of increased ascorbate (~2.5–3.5 µmol g^–1^ FW) in PLP mutants of tomato and Arabidopsis (Aarabi *et al.*, 2023; Bournonville *et al.*, 2023). Similarly, knockout or overexpression of PLP in soybean modestly increased or decreased ascorbate, respectively (Zhang *et al.*, 2023). Critically, binding of PLP to GGP occurs *in vivo* and is disrupted by blue light (Aarabi *et al.*, 2023), and this binding inhibits enzyme activity of a recombinant GGP (Bournonville *et al.*, 2023). Physical association between sequential enzymes of the d-Man/l-Gal pathway enzymes from GMP through to l-GalDH is suggested from co-immunoprecipitation and gel filtration experiments (Fenech *et al.*, 2021). Association of enzymes into ‘metabolons’ can sometimes improve or direct flux (Sweetlove and Fernie, 2018), but more work is needed to assess the functional significance in the d-Man/l-Gal pathway. Many other factors will influence ascorbate synthesis, and transcription factors controlling ascorbate accumulation via expression of GGP and other d-Man/l-Gal pathway enzymes are being identified (Broad *et al.*, 2020b; Liu *et al.*, 2022, 2023; Xu *et al.*, 2023; Zhang *et al.*, 2023). Jasmonic acid and methyl jasmonate increase ascorbate concentration and GGP/GME expression in Arabidopsis liquid-cultured seedlings and cell suspension cultures (Sasaki-Sekimoto *et al.*, 2005; Wolucka *et al.*, 2005) for up to 48 h after application, along with AtGGP1/VTC2, ATGGP2/VTC5, and GME expression. In Arabidopsis, publicly available transcriptome data indicate that GGP2 expression is specifically responsive to ozone and the PAMP flg22 additionally to light.

## Metabolic engineering and biotechnology

As well as improving nutritional value, increased ascorbate might improve stress resistance. Discovery of the d-Man/l-Gal pathway resulted in a flurry of patent applications related to the use of GME, l-GalDH, and l-GalLDH in engineering plants for increased ascorbate and the possibility that l-GalDH, as a plant-specific enzyme, could be a herbicide target (Bauw *et al.*, 1998; Berry *et al.*, 1999; Smirnoff and Wheeler, 1999). The increasingly detailed understanding of the d-Man/l-Gal pathway and particularly the complex control of GGP activity (Fig. 4) will inform metabolic engineering strategies to increase ascorbate in specific tissues in a controlled manner. The identification of translational control by the GGP uORF, as noted in the previous section, has already provided a simple route to increasing ascorbate via gene editing. However, this approach has also shown that producing too much ascorbate can be damaging to development and fertility in tomato (Deslous *et al.*, 2021). This effect seems to extend to Arabidopsis, where overexpression of GGP1 with a pollen-specific promoter decreases pollen production and growth (Weigand *et al.*, 2023). However, in this case, pollen ascorbate was not increased, suggesting that increased production of degradation products or diversion of GDP-sugars from growth-critical glycosylation reactions could be the reason (Fig. 3). GME mutants in tomato and Arabidopsis have impaired pollen growth and fertility which is not rescued by ascorbate, indicating a critical role for GPP-sugars in pollen function (Mounet-Gilbert *et al.*, 2016; Qi *et al.*, 2017). Furthermore, the *tdf1* Arabidopsis mutant has decreased expression of an ascorbate oxidase-like protein, has double wild-type ascorbate in its inflorescences, and does not develop pollen normally (Wu *et al.*, 2023) (more details are provided in the next section). Clearly, the reason for the deleterious effect of very high ascorbate requires further investigation (Castro *et al.*, 2018). Transgenic approaches to increasing ascorbate by overexpression of d-Man/l-Gal pathway enzymes have been well reviewed and are not covered in detail here (Ishikawa *et al.*, 2006; Macknight *et al.*, 2017; Broad *et al.*, 2020b; Terzaghi and De Tullio, 2022; Castro *et al.*, 2023). As noted in the previous section, overexpression of GGP tends to have the greatest effect. Another approach to metabolic engineering is to introduce or boost routes to l-GalL or l-GulL production via d-galacuronate or d-glucuronate, respectively, as analogues of the protist and animal pathways (Smirnoff, 2003; Wheeler *et al.*, 2015). Overexpression of strawberry d-galacturonate reductase in Arabidopsis increases leaf ascorbate 2- to 3-fold (Agius *et al.*, 2003). More controversially, increasing d-glucuronate production by overexpressing *myo*-inositol oxygenase has been reported to increase (Lorence *et al.*, 2004) or not affect (Endres and Tenhaken, 2009) ascorbate in Arabidopsis.

Very large amounts of ascorbate are manufactured for vitamin supplements for human and fish diets, and for food/beverage manufacturing as an antioxidant preservative, so there is great interest in engineering microorganisms for ascorbate synthesis (Hancock and Viola, 2002; Running *et al.*, 2004; Wang *et al.*, 2018). The dominant Reichstein process, which has multiple chemical steps and one microbial conversion, is highly optimized to convert glucose to ascorbate. This is important because the price differential between precursor and product is small. A one-step fermentation to manufacture ascorbate in bacteria or yeast could be superior and cleaner. Many of the introduced pathways are synthetic, often aimed at producing the 2-keto-l-gulonate as the precursor. The existing yeast d-erythroascorbate pathway can utilize the plant intermediates l-Gal and l-GalL, which provides a useful starting point (Hancock *et al.*, 2000; Sauer *et al.*, 2004; Branduardi *et al.*, 2007). Encouragingly, the entire plant d-Man/l-Gal pathway from Glc has been successfully reconstituted in *Escherichia coli* (Tian *et al.*, 2022) and *Saccharomyces cerevisiae* (Zhou *et al.*, 2021), although current yields are likely to be too low for commercial use.

## The functions of ascorbate

### Using Arabidopsis *vtc* mutants to understand the functions of ascorbate

The *A. thaliana vtc* mutants have been invaluable in elucidating ascorbate biosynthesis and, except for VTC3, their roles are now well established. Understandably, many researchers have been drawn to use these mutants to investigate the functions of ascorbate, particularly in relation to photosynthesis, photoprotection, pathogen response, and abiotic stresses (summarized in Supplementary Table S1). Complete knockout of ascorbate-specific biosynthesis genes (GGP onwards) is lethal. The exception is VTC4, whose phosphatase activity is not specific to l-Gal 1-P and because other enzymes with similar catalytic activity are present and enable ascorbate synthesis, albeit resulting in lower concentration (Conklin *et al.*, 2006). l-GalLDH mutants are also affected in mitochondrial Complex 1 formation as discussed in the biosynthesis section. The *vtc1* mutants are not just affected in ascorbate biosynthesis because GDP-Man is also needed for cell wall polysaccharide synthesis and protein glycosylation as previously discussed. The function of VTC3 is unknown, so *vtc3* mutants could be pleiotropic. This leaves GGP mutants (*vtc*2/5), the first step dedicated to ascorbate production, and l-GalDH as the most appropriate to use for investigating ascorbate function. The original EMS mutants of GGP (*vtc2-1*, *2-2*, and *2-3*) (Conklin *et al.*, 2000; Jander *et al.*, 2002) were important in pathway identification. Of these, *vtc2*-*1* and *vtc2-2* have smaller rosettes than the wild type, leading to speculation that reduction of ascorbate to ~20% of the wild-type concentration affects growth and flowering (Pavet *et al.*, 2005; Barth *et al.*, 2006; Olmos *et al.*, 2006; Kotchoni *et al.*, 2009; Kerchev *et al.*, 2011). However, the identification of insertion mutants (*vtc2-4* and *vtc2-5*) in *VTC2* which have similarly low ascorbate but are only slightly smaller, along with finding that backcrossing *vtc2-1* to the wild type segregated small size from ascorbate deficiency (Lim *et al.*, 2016), confirms that severely decreased growth in this mutant is not linked to ascorbate deficiency. A comparison of *vtc2-1* and *vtc2-4* showed smaller rosette biomass in both mutants in one study (Plumb *et al.*, 2018) but no difference in another (Lim *et al.*, 2016). The take-home message is that the *vtc* mutants should be used very carefully for assessing the functions of ascorbate, and of course it is very likely that observed phenotypes are highly dependent on the environment. The critical growth maintenance functions of ascorbate in plants will need mutants containing less than ~20% of wild-type ascorbate concentration since these plants can grow, while plants with no ascorbate are unable to grow (Dowdle *et al.*, 2007; Lim *et al.*, 2016; Fenech *et al.*, 2021). Meanwhile, either *vtc2-4* or *vtc2-5* should be used, or the same phenotype should be observed in mutants from different steps in the pathway before it is attributed to ascorbate. Decreased NPQ and increased basal pathogen resistance fall into this well-supported category (Supplementary Table S1).

### Antioxidant

Probably the greatest focus on ascorbate in plants has been on its antioxidant role. As noted in the Introduction, it is an effective remover of H_2_O_2_ (catalysed by the plant-specific enzyme APX) and radicals. Consequently a range of *vtc* and *apx* mutants contain more H_2_O_2_ (Mukherjee *et al.*, 2010), potentially influencing H_2_O_2_ signalling (Smirnoff and Arnaud, 2019; Mittler *et al.*, 2022) and stress responses. With the caveat that in some cases only one mutant has been investigated, Arabidopsis *vtc* mutants are generally more susceptible to a range of abiotic stresses that are presumed to increase ROS or radical production such as ozone, sulfur dioxide, UV-B and C radiation, high salinity, and temperature extremes (Conklin *et al.*, 1996; Smirnoff, 2000; Conklin and Barth, 2004; Huang *et al.*, 2005; Larkindale *et al.*, 2005; Gao and Zhang, 2008; Wang *et al.*, 2012; Yao *et al.*, 2015; Hoang *et al.*, 2021b). It is therefore assumed that more ascorbate will improve abiotic stress resistance, and numerous metabolic engineering attempts have this goal. Improved stress tolerance is often claimed, although the experimental conditions may not be relevant to field conditions (Smirnoff, 2018). Results are summarized by Broad *et al.* (2020a, b). A wide range of *vtc* mutants have increased basal resistance to biotrophic pathogens such as *Hyaloperonospora parasitica* and *Pseudomonas syringae*, along with increased expression of various pathogen response genes, and SA and camalexin accumulation (Pastori *et al.*, 2003; Barth *et al.*, 2004; Colville and Smirnoff, 2008; Mukherjee *et al.*, 2010). It is proposed that increased H_2_O_2_ induces SA-dependent defence (Mukherjee *et al.*, 2010). GGP2 expression and a small increase in ascorbate is induced 60–90 min after elicitation of Arabidopsis cell cultures with harpin (Czobor *et al.*, 2017). The significance in terms of ascorbate concentration and interaction with pathogens is unknown. In contrast, *vtc1* and *vtc2-1* are more sensitive to the necrotrophic fungus *Alternaria brassicicola* (Botanga *et al.*, 2012). It is therefore possible that ascorbate concentration is a balance between the need for antioxidant defence, photosynthesis, and pathogen resistance.

### Photosynthesis and photoprotection

The involvement of ascorbate in photosynthesis was recognized some time ago (Marrè and Arrigoni, 1958; Marrè *et al.*, 1959; Mapson, 1964). Its functions in chloroplasts are evident considering its responsiveness to light via GGP activity. These functions are: (i) the removal of H_2_O_2_ produced by oxygen photoreduction at PSI using thylakoid and stromal APXs and (ii) energy dissipation via the xanthophyll cycle (NPQ) in which thylakoid lumen VDE requires ascorbate as a substrate. Other proposed roles include as an emergency electron donor to PSII and inactivation of the oxygen-evolving complex under some conditions. Ascorbate may regenerate tocopherol from tocopheroxyl radicals resulting from singlet oxygen production in PSII. These roles in photosynthesis and photoprotection have been well reviewed and so are not considered in detail here (Foyer and Shigeoka, 2011; Foyer, 2018; Toth, 2023).

### Growth and development

The role of ascorbate in growth and development requires deeper analysis. It seems likely that its key functions are not readily evident from the *vtc* mutants. Ascorbate disappears from seeds during maturation and desiccation, and growth following imbibition is coincident with ascorbate accumulation (Arrigoni *et al.*, 1992, 1997; Tommasi *et al.*, 1999; Pallanca and Smirnoff, 2000; De Tullio and Arrigoni, 2003). Fully ascorbate-deficient mutants germinate, but growth is then arrested (Lim *et al.*, 2016; Fenech *et al.*, 2021). Further investigation of which processes are impeded in these seedlings is needed, possibilities being the antioxidant role (i.e. ROS removal to maintain a suitable redox state for cell division) (Potters *et al.*, 2004; Schnaubelt *et al.*, 2015), iron mobilization, or a requirement of 2-ODDs for growth or epigenetic regulation. In roots, the quiescent centre cells have a low rate of cell division. Cell division is influenced by highly oxidized ascorbate and GSH pools which are proposed to arrest transition from the G_1_ to S phase of cell division. A model in which indole-3-acetic acid (IAA) induces AO activity in the QC to locally oxidize ascorbate is proposed and, intriguingly, AO could also decarboxylate IAA (Kerk and Feldman, 1995; Kerk *et al.*, 2000; Jiang *et al.*, 2003). These results reflect the wider picture of dependence of cell division on the redox state of ascorbate and GSH (Potters *et al.*, 2004; Schnaubelt *et al.*, 2015). Recent work indicates that ascorbate status could also influence the differentiation of tapetal cells for pollen production. Normal tapetal development and pollen formation in Arabidopsis requires the transcription factor DEFECTIVE IN TAPETAL DEVELOPMENT AND FUNCTION 1 (TDF1) to control the transition from cell division to differentiation. TDF1 increases expression of a copper oxidase SKS18 (in the same family as AO) and, at the same time, represses GMP (*VTC1*) expression (Wu *et al.*, 2023). The authors present a model in which low ascorbate, resulting from its destruction by AO activity of SKS118 and decreased synthesis due to lower GMP activity, encourages a switch from cell division to differentiation, possibly via increased ROS production. While the evidence is compelling, two points require clarification. Firstly, although it is feasible that SKS18 has AO activity, the recombinant protein was inactive until Cu^2+^ was added to the assay. Unfortunately, this evidence is weak because Cu^2+^ alone catalytically oxidizes ascorbate (Shen *et al.*, 2021) and, since SKS18 is likely to be a secreted glycoprotein, it will not in any case be processed correctly in *E. coli.* Therefore, although SKS18 could well have AO activity, the results are equivocal. Secondly, disruption of GDP-Man synthesis could itself affect differentiation.

### Ascorbate and iron

**Fig. 6.**
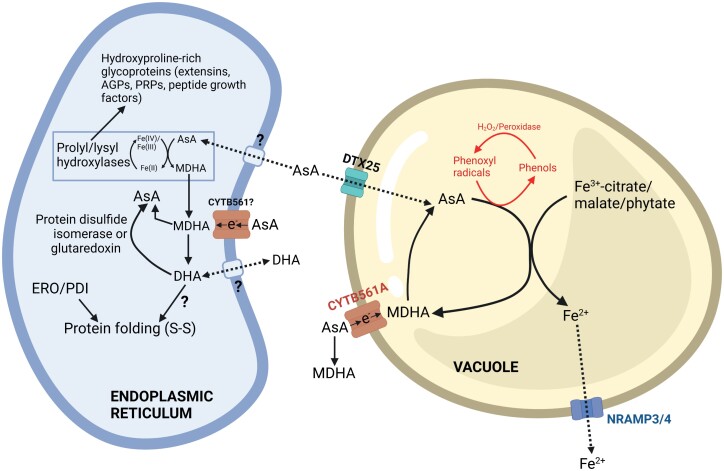
The role of ascorbate in iron-related processes. Ascorbate is required to prevent irreversible over-oxidation of Fe in 2-oxoglutarate-dependent dioxygenase (2-ODD) enzymes. 2-ODDs hydroxylating prolyl and lysyl residues of glycoproteins (e.g. prolyl 4-hydroxylase) are located in the ER. Inhibition or knockout of ER-localised prolyl 4-hydroxylases increases ascorbate concentration, suggesting that 2-ODD protection in the ER is a significant sink for ascorbate. Nothing is known about uptake of ascorbate or DHA, or the processes involved in reduction of MDHA or DHA in this compartment, but possible routes are shown. DHA could be reduced by GSH using PDI or glutaredoxin or MDHA could be reduced *via* cytc b_5_ mediated transmembrane electron transport. PDI is also involved in protein folding by disulfide bond formation, along with the H_2_O_2_-generating endoplasmic reticulum oxidoreductin (ERO). DHA could also facilitate disulfide bond formation. The recent identification of a tonoplast ascorbate transporter (DTX25) and transmembrane electron transporter (CYTB561A) provides a mechanism for moving ascorbate into the vacuole and regenerating MDHA. This system facilitates Fe mobilisation from vacuoles during seed germination and aids vacuolar H_2_O_2_ scavenging by type III peroxidases. Created with BioRender.com. Abbreviations: AGP, arabinogalactan protein; AsA, ascorbate; DHA, dehydroascorbate; ERO, endoplasmic reticulum oxidoreductin; P4H, prolyl 4-hydroxylase; MDHA, monodehydroascorbate; NRAMP, Natural resistance-associated macrophage protein; PDI, protein disulfide isomerase; PRP, proline-rich protein.

Ascorbate can both chelate Fe^3+^ and readily reduce it to the more soluble Fe^2+^ (also Cu^2+^ to Cu^+^) and there is evidence that it has a pervasive role in Fe uptake, transport, and storage in animals, as well as being a cofactor/chaperone to prevent Fe over-oxidation in 2-ODDs (Lane and Richardson, 2014; Badu-Boateng and Naftalin, 2019; De Tullio, 2020). While the evolution of ascorbate in eukaryotes could have been driven by protection against ROS arising in the great oxygenation event (GOE) started by cyanobacterial oxygenic photosynthesis (Gest *et al.*, 2013), the availability of soluble Fe^2+^ also plummeted following the GOE. It is perhaps not a coincidence that ascorbate is effective in Fe^3+^ reduction and is also an antioxidant dealing with ROS and minimizing hydroxyl radicals formed, for example, by interaction of Fe^2+^ and H_2_O_2_ (the Fenton reaction). Thus, ascorbate could have played a role in both maintaining Fe supply and protecting against its pro-oxidant effects. Indeed, it has been proposed that the appearance of ascorbate was an important factor in the development of multicellular organisms because of its antioxidant role but also in the function of 2-ODDs in extracellular matrix protein synthesis and epigenetic control of stem cell activity (Edgar, 2019). It is striking that ascorbate accumulation is massively increased by Fe deficiency and H_2_O_2_ in *Chlamydomonas* (Urzica *et al.*, 2012a, b). This response is much greater than in vascular plants (Zaharieva *et al.*, 1999; Zaharieva and Abadia, 2003) where the basal ascorbate concentration is much higher. Feeding ascorbate (or GSH) to Arabidopsis seedlings alleviates Fe-deficiency chlorosis (Ramirez *et al.*, 2013). Embryos of ascorbate-deficient Arabidopsis mutants (*vtc2-4* and *vtc5*) have low Fe (Grillet *et al.*, 2014). Recently, a tonoplast-located ascorbate transporter in the MATE family (AtDTX25) has been identified. A knockout mutant has less ascorbate and more Fe in isolated vacuoles and is more sensitive to Fe deficiency than the wild type (Hoang *et al.*, 2021a). These observations provide a model for mobilization of Fe in germinating seedlings which is stored as vacuolar Fe^3+^-citrate, malate, or phytate chelates (Fig. 6). Ascorbate is transported into the vacuole where it reduces Fe^3+^, and the resulting Fe^2+^ is transported to the cytosol via tonoplast natural resistance-associated macrophage protein (NRAMP) transporters (Bastow *et al.*, 2018; Hoang *et al.*, 2021a). Plasma membrane-localized Cyt *b* can reduce MDHA using ascorbate as electron donor (Horemans *et al.*, 1994), although evidence for its role *in vivo* has been lacking. Recently a tonoplast Cyt *b*_561_ able to reduce vacuolar MDHA using cytosolic ascorbate has been identified (Gradogna *et al.*, 2023). This activity could contribute to regeneration of vacuolar ascorbate from MDHA (Fig. 6). In summary, ascorbate is involved in Fe transport into seeds and its mobilization during germination. Possibly the arrested germination of fully ascorbate-deficient Arabidopsis mutants (Dowdle *et al.*, 2007; Lim *et al.*, 2016; Fenech *et al.*, 2021) could be caused in part by impaired Fe mobilization. Further evidence for a connection between ascorbate and Fe is related to a connection with response to phosphorus deficiency. Fe deficiency decreases ascorbate in Arabidopsis seedlings and causes chlorosis. Chlorosis is reversed by ascorbate supplementation in the wild type but not in a mutant of the PHT4:4 chloroplast ascorbate transporter. Additionally VTC1, VTC2, and VTC4 expression is induced by combined Fe and P deficiency, and ascorbate concentration is maintained and chlorosis does not develop, except in *pht4:4* and *vtc4* mutants (Nam *et al.*, 2021). These intriguing results indicate links between ascorbate, Fe, and P nutrition and photosynthesis which require further investigation.

The foundational biomedical interest in ascorbate came from scurvy, the severe ascorbate deficiency disease. This is largely characterized by symptoms related to defective connective tissue because of collagen deficiency. Collagen contains hydroxyproline and hydroxylysine residues required for its function and formed post-translationally by prolyl 4-hydroxylases (P4Hs) and lysyl hydroxylases in the ER (Fig. 6). These are 2-ODDs and require ascorbate to prevent overoxidation of their active site Fe. Prolyl hydroxylase is inactivated after only 15–30 reaction cycles in the absence of ascorbate and other reductants (including thiols) are much less effective at maintaining activity (Myllylä *et al.*, 1978, 1984; Majamaa *et al.*, 1986). Plants contain many 2-ODDs (~100 in Arabidopsis) which are involved in diverse processes (Kawai *et al.*, 2014). ER-localized 2-ODDs hydroxylate prolyl and lysyl residues in extracellular structural proteins of the hydroxyproline-rich glycoprotein family such as extensins, proline-rich proteins, and arabinogalactan proteins (AGPs). Also, various peptide hormones are hydroxylated by unknown P4Hs (Stuhrwohldt and Schaller, 2019; Stuhrwohldt *et al.*, 2020), while others, mostly cytosolic, are involved in synthesis and metabolism of hormones (ethylene, gibberellin, and IAA) and specialized/secondary metabolites (Smirnoff, 2018; De Tullio, 2020). Ascorbate function in mammals has experienced greatly increased interest following evidence that its concentration might influence the activity of demethylases in the 2-ODD family involved in DNA demethylation [ten eleven translocation (TET) proteins] and histone demethylation (Jumonji C-domain-containing demethylases), thereby influencing epigenetic events and processes such as stem cell differentiation (Young *et al.*, 2015; Agathocleous *et al.*, 2017; Cimmino *et al.*, 2017). Ascorbate is a substrate for a *Chlamydomonas* TET homologue (CMD1) in the place of 2-oxoglutarate. It donates a glyceryl group to 5mC with formation of glyoxylate and carbon dioxide (Xue *et al.*, 2019; Li *et al.*, 2021). A CRISPR-generated knockout of CDM1 caused high light sensitivity and altered expression of various photosynthesis- and photoprotection-related genes. It will be interesting to know if this type of TET protein is more widespread, although proteins with substantial sequence similarity seem limited to a few green algae.

Ascorbate deficiency in humans and guinea pigs impairs activity of 2-ODDs, including P4H and TET. Therefore, are ascorbate-deficient plants affected in 2-ODD activity? This question was assessed by measuring the hydroxyproline content of *vtc1* and *vtc2-1.* They do not have decreased hydroxyproline in cell wall proteins (Sultana *et al.*, 2015) which suggests that the 20–30% of wild-type ascorbate in these mutants can maintain prolyl hydroxylase activity. Nevertheless, there are two lines of evidence suggesting that ascorbate has a major role in 2-ODDs. Firstly, inhibition of prolyl hydroxylase with 3,4-dl-dehydroproline increases ascorbate concentration in several plant species (De Gara *et al.*, 1991; De Tullio *et al.*, 1999). Secondly, a number of Arabidopsis 2-ODD mutants, including prolyl hydroxylases, have increased ascorbate (Mahmood and Dunwell, 2020). These observations suggest the ER-localized 2-ODDs are a major sink for ascorbate, and regeneration in the ER is not sufficient to prevent loss following oxidation. Little is known about ascorbate in the ER lumen of plants (Fig. 6). Immunocytochemical measurement of ascorbate shows that it is present in most cell compartments, including the vacuole, but staining is not seen in the ER or Golgi lumen (Zechmann *et al.*, 2011), possibly because it is predominantly present as DHA. The ER is a relatively ‘oxidizing’ compartment to favour formation of protein disulfides common in extracellular proteins and, using a roGFP GSH/GSSG sensor functional in the ER (Grx1–roGFP2iL–HDEL), the GSH/GSSG ratio is 100 times smaller in the ER than in the cytosol (Ugalde *et al.*, 2022). This observation supports the view that any ER localized MDHA or DHA reducing systems have limited capacity. MDHAR and DHAR are not predicted to be present in the ER, but protein disulfide isomerase (PDI) and glutaredoxin (GRX) are both present (Ugalde *et al.*, 2022) and have DHAR activity (Wells *et al.*, 1990). In Arabidopsis, a Cyt *b*_561_ (At5g38630) is predicted to be in the ER membrane. Since a tonoplast Cyt *b*_561_ can reduce MDHA using ascorbate (Gradogna *et al.*, 2023), ER lumen MDHA could also be reduced to ascorbate via this electron transporter. It is not known how ascorbate (or DHA) is transported across the ER membrane.

Considering the evidence for consumption (and loss) of ascorbate in the ER due to 2-ODD activity, and the large demand for hydroxylated extracellular proteins such as extensin and AGPs during cell expansion, it is likely that growth arrest in ascorbate null mutants could be caused by limited 2-ODD activity while the 80–90% decrease in ascorbate in the *vtc* mutants is still sufficient to support 2-ODD activity. Overall, the role of ascorbate in Fe uptake and 2-ODD function deserves further investigation. In addition to Fe-related roles, it has been proposed in mammals that DHA could participate in protein folding via S–S formation along with endoplasmic reticulum oxidoreductase (ERO). Indeed, ascorbate deficiency in mammalian systems is suggested to contribute to ER stress (Szarka and Lorincz, 2014).

## Conclusions

The biosynthetic pathway of ascorbate through GDP-Man/l-Gal (Wheeler *et al.*, 1998) has been supported by subsequent evidence from ascorbate-deficient Arabidopsis mutants and by knockdowns in other species. The pathway is specific to the green plant lineage (Wheeler *et al.*, 2015). The first dedicated enzyme of the pathway, GGP, funnels GDP-sugars away from their functions in protein mannosylation and glycan synthesis. Recent work has shown it is the strongest controlling step in the pathway, with its expression being the result of transcriptional and translational controls along with light-dependent interaction with a PAS-LOV protein. The translational control through a uORF could enable feedback inhibition of ascorbate synthesis. This mechanism is providing a prototypical example of the application of gene editing to biofortification, but has also revealed potential negative effects on development and fertility, particularly pollen function. The reason for this requires further investigation by unravelling direct effects of ascorbate from perturbed GDP-sugar metabolism. The mechanism of ribosome stalling on the uORF and the effect of ascorbate on this process is under investigation. GGP is usually encoded by two paralogous genes in vascular plants. Expression of both is increased by high light in Arabidopsis, but one is also responsive to jasmonate and pathogen-associated molecular patterns (PAMPs), suggesting a division of labour. Signalling mechanisms involved in light induction need to be clarified along with the specific function of the GGP isoforms. The last step of the pathway using l-GalLDH occurs in the intermembrane space of mitochondria. Indeed, a unique feature of plant mitochondria is the dual use of l-GalLDH in Complex 1 assembly and in catalysing the oxidation of l-GalL to ascorbate via an interaction with Cyt *c*.

Ascorbate-deficient mutants have been used to reveal roles of ascorbate in photoprotection and antioxidant defence, although care in interpretation is needed because of pleiotropic effects of many of the mutations in d-Man/l-Gal pathway genes. Despite increased stress sensitivity (and activation of basal pathogen defence), Arabidopsis mutants with ~20% normal ascorbate have few major growth defects, while full deficiency is lethal. This is puzzling given the apparently tight control of the ascorbate concentration by GGP, so possibly ‘excess’ ascorbate is needed to deal with fluctuating environmental conditions. Understanding the basal functions of ascorbate needs plants with lower ascorbate. Based on the emerging evidence, we predict that very low ascorbate will more obviously impact two iron-related processes: activity of 2-ODDs, particularly in the ER; and iron trafficking. Ascorbate is an effective reducer of higher oxidation states of Cu and Fe, thereby improving availability, and it is an antioxidant. It can be proposed that this dual capacity was the driver for the evolution of ascorbate biosynthesis capacity though diverse pathways in eukaryotes as a response to increased oxygen and decreased Fe^2+/^Cu^+^ availability following the appearance of oxygenic photosynthesis in cyanobacteria.

## Supplementary data

The following supplementary data are available at *JXB* online.

Table S1. Growth, development, and stress responses of *Arabidopsis thaliana* ascorbate-deficient (*vtc*) mutants.

erad505_suppl_Supplementary_Table_S1

erad505_suppl_Supplementary_Table_S2

## Data Availability

No new data are included in this review.
