# Supplementary material for: The ascorbate biosynthesis pathway in plants is known, but there is a way to go with understanding control and functions
Source: J Exp Bot. 2024 Feb 1;75(9):2604–30. doi: 10.1093/jxb/erad505 (PMC11066809; doi:10.1093/jxb/erad505)
Supplement: erad505_suppl_Supplementary_Table_S1 [file erad505_suppl_supplementary_table_s1.pdf]

Supplementary Table S1. Growth, development and stress responses of *Arabidopsis thaliana* ascorbate deficient (*vtc*) mutants.

A reasonably comprehensive list reports on the characteristics of *vtc* mutants are listed below. Readers are advised to check individual references for details of mutants used in each study, specific phenotypes and experimental conditions.

Condition	Mutants/alleles	Effect	References
Photosynthesis and high light response.	All	Generally, more sensitive to high light. Decreased NPQ and zeaxanthin formation in high light. Slower anthocyanin accumulation in high light.	(Giacomelli *et al*., 2006; Havaux *et al*., 2006; Kanwischer *et al*., 2005; Muller-Moulé *et al*., 2002; Muller-Moulé *et al*., 2004; Muller-Moulé *et al*., 2003; Page *et al*., 2012; Plumb *et al*., 2018; Podmaniczki *et al*., 2021; Smirnoff, 2000; Toth *et al*., 2011; Toth *et al*., 2009; Wormuth *et al*., 2006; Zeng *et al*., 2018)
UV-C	*vtc1*	Increased double strand DNA breaks	(Filkowski *et al*., 2004)
UV-B	*vtc1-1, vtc2*	Increased sensitivity	(Gao and Zhang, 2008; Yao *et al*., 2015)
Ozone/SO_2	*vtc1-1, vtc2-1/2-2/2-3, vtc2-1, vtc4-1*	Increased ozone sensitivity (except *vtc2-2*). *vtc1* is SO_2 sensitive (others not reported)	(Conklin *et al*., 2000; Conklin *et al*., 1996)
Temperature extremes	*vtc1-1, vtc2-1*	Decreased basal thermotolerance. Protection against PSII inactivation. Increased chilling sensitivity.	(Larkindale *et al*., 2005; Toth *et al*., 2011; Wang *et al*., 2012)
High salinity (NaCl)	*vtc2-4, vtc2-5, vtc1-1, vtc2-1, vtc3-1, vtc4-1*	More sensitive	(Hoang *et al*., 2021; Huang *et al*., 2005; Koffler *et al*., 2014a; Smirnoff, 2000)
Drought and osmotic stress	*vtc2-4, vtc2-5, vtc1-1, vtc2-1, vtc3-1, vtc4-1* and GalLDH knockdown	More sensitive; reduced stomatal aperture. More ABA; increased lipid oxidation, decreased tocopherol/β-carotene	(Brossa *et al*., 2011; Hoang *et al*., 2021; Koffler *et al*., 2014a; Li *et al*., 2014; Lopez-Carbonell *et al*., 2006; Munne-Bosch and Alegre, 2002)
Cu or Cd toxicity	vtc2-*	Tolerance unaffected or affected *via* altered GSH	(Hoang *et al*., 2021; Jozefczak *et al*., 2015; Koffler *et al*., 2014b; Peto *et al*., 2013)
Pathogen resistance (biotrophs)	*vtc1-1, vtc2-1, vtc2-2; vtc3-1, vtc4-1*	Increased NPR1-mediated basal resistance to *Pseudomonas syringae* and *Hyaloperonospora via* H_2O_2-induced salicylic acid accumulation. Greater expression of PR proteins. Increased hypersensitive-like lesions	(Barth *et al*., 2004; Brosché and Kangasjärvi, 2012; Colville and Smirnoff, 2008; Mukherjee *et al*., 2010; Pastor *et al*., 2013; Pastori *et al*., 2003; Pavet *et al*., 2005)
Pathogen resistance (necrotrophs)	*vtc1-1, vtc2-1*	More susceptible to *Alternaria*	(Botanga *et al*., 2012)
Ammonium hypersensitivity	*vtc1*	Root growth more inhibited by ammonium in a VTC1 specific manner.	(Barth *et al*., 2010; Qin *et al*., 2008; Zhang *et al*., 2021)
Growth and development	*vtc1-1; vtc 2-1, 2-2, 2,4, 2-5*	Growth (rosette size) slightly less or unaffected. Early senescence of older leaves. Altered hormone responses. Flowering time accelerated.	(Barth *et al*., 2004; Caviglia *et al*., 2018; Dowdle *et al*., 2007; Kerchev *et al*., 2011; Kka *et al*., 2018; Kotchoni *et al*., 2009; Li *et al*., 2016; Lim *et*

		Total ascorbate deficiency in *vtc2/vtc5* double mutants arrests growth after germination. Very small size of *vtc2-1* caused by a second site mutation. *vtc1* growth defects could be related to impaired GDP-mannose production. Altered cell wall glycoproteome.	*al.*, 2016; Olmos *et al.*, 2006; Plumb *et al.*, 2018; Sultana *et al.*, 2015; Veljovic-Jovanovic *et al.*, 2001)
Iron nutrition	*vtc2-4, vtc5-1, vtc5-2, vtc4-1*	Decreased iron in seeds. Iron deficiency chlorosis in *vtc4* during P deficiency	(Grillet *et al.*, 2014; Nam *et al.*, 2021)
Hydrogen peroxide concentration	*vtc1-1, vtc2-1, vtc3-1, vtc4-1*	Increased	(Heyneke *et al.*, 2013; Mukherjee *et al.*, 2010; Naydov *et al.*, 2010)

References

Barth C, Gouzd ZA, Steele HP, Imperio RM. 2010. A mutation in GDP-mannose pyrophosphorylase causes conditional hypersensitivity to ammonium, resulting in Arabidopsis root growth inhibition, altered ammonium metabolism, and hormone homeostasis. Journal of Experimental Botany **61**, 379-394.

Barth C, Moeder W, Klessig DF, Conklin PL. 2004. The timing of senescence and response to pathogens is altered in the ascorbate-deficient Arabidopsis mutant *vitamin C-1*. Plant Physiol **134**, 1784-1792.

Botanga CJ, Bethke G, Chen Z, Gallie DR, Fiehn O, Glazebrook J. 2012. Metabolite profiling of Arabidopsis inoculated with *Alternaria brassicicola* reveals that ascorbate reduces disease severity. Molecular Plant-Microbe Interactions **25**, 1628-1638.

Brosché M, Kangasjärvi J. 2012. Low antioxidant concentrations impact on multiple signalling pathways in *Arabidopsis thaliana* partly through NPR1. Journal of Experimental Botany **63**, 1849-1861.

Brossa R, Lopez-Carbonell M, Jubany-Mari T, Alegre L. 2011. Interplay Between Abscisic Acid and Jasmonic Acid and its Role in Water-oxidative Stress in Wild-type, ABA-deficient, JA-deficient, and Ascorbate-deficient Arabidopsis Plants. Journal of Plant Growth Regulation **30**, 322-333.

Caviglia M, Mazorra Morales LM, Concellon A, Gergoff Grozeff GE, Wilson M, Foyer CH, Bartoli CG. 2018. Ethylene signaling triggered by low concentrations of ascorbic acid regulates biomass accumulation in *Arabidopsis thaliana*. Free Radical Biology and Medicine **122**, 130-136.

Colville L, Smirnoff N. 2008. Antioxidant status, peroxidase activity, and PR protein transcript levels in ascorbate-deficient *Arabidopsis thaliana vtc* mutants. Journal of Experimental Botany **59**, 3857-3868.

Conklin PL, Saracco SA, Norris SR, Last RL. 2000. Identification of ascorbic acid-deficient *Arabidopsis thaliana* mutants. Genetics **154**, 847-856.

Conklin PL, Williams EH, Last RL. 1996. Environmental stress sensitivity of an ascorbic acid-deficient Arabidopsis mutant. Proceedings of the National Academy of Sciences of the United States of America **93**, 9970-9974.

Dowdle J, Ishikawa T, Gatzek S, Rolinski S, Smirnoff N. 2007. Two genes in *Arabidopsis thaliana* encoding GDP-L-galactose phosphorylase are required for ascorbate biosynthesis and seedling viability. Plant Journal **52**, 673-689.

Filkowski J, Kovalchuk O, Kovalchuk I. 2004. Genome stability of *vtc1*, *tt4*, and *tt5 Arabidopsis thaliana* mutants impaired in protection against oxidative stress. Plant Journal **38**, 60-69.

Gao Q, Zhang LX. 2008. Ultraviolet-B-induced oxidative stress and antioxidant defense system responses in ascorbate-deficient *vtc1* mutants of *Arabidopsis thaliana*. Journal of Plant Physiology **165**, 138-148.

Giacomelli L, Rudella A, van Wijk KJ. 2006. High light response of the thylakoid proteome in Arabidopsis wild type and the ascorbate-deficient mutant *vtc2-2*. A comparative proteomics study. Plant Physiol **141**, 685-701.
Grillet L, Ouerdane L, Flis P, Hoang MTT, Isaure MP, Lobinski R, Curie C, Mari S. 2014. Ascorbate efflux as a new strategy for iron reduction and transport in plants. Journal of Biological Chemistry **289**, 2515-2525.
Havaux M, Triantaphylides C, Genty B. 2006. Autoluminescence imaging: a non-invasive tool for mapping oxidative stress. Trends in Plant Science **11**, 480-484.
Heyneke E, Luschin-Ebengreuth N, Krajcer I, Wolkinger V, Muller M, Zechmann B. 2013. Dynamic compartment specific changes in glutathione and ascorbate levels in Arabidopsis plants exposed to different light intensities. BMC Plant Biology **13**, 104.
Hoang MTT, Doan MTA, Nguyen T, Tra DP, Chu TN, Dang TPT, Quach PND. 2021. Phenotypic characterization of Arabidopsis ascorbate and glutathione deficient mutants under abiotic stresses. Agronomy-Basel **11**, 764.
Huang CH, He WL, Guo JK, Chang XX, Su PX, Zhang LX. 2005. Increased sensitivity to salt stress in an ascorbate-deficient Arabidopsis mutant. Journal of Experimental Botany **56**, 3041-3049.
Jozefczak M, Bohler S, Schat H, Horemans N, Guisez Y, Remans T, Vangronsveld J, Cuypers A. 2015. Both the concentration and redox state of glutathione and ascorbate influence the sensitivity of arabidopsis to cadmium. Annals of Botany **116**, 601-612.
Kanwischer M, Porfirova S, Bergmuller E, Dormann P. 2005. Alterations in tocopherol cyclase activity in transgenic and mutant plants of arabidopsis affect tocopherol content, tocopherol composition, and oxidative stress. Plant Physiol **137**, 713-723.
Kerchev PI, Pellny TK, Vivancos PD, Kiddle G, Hedden P, Driscoll S, Vanacker H, Verrier P, Hancock RD, Foyer CH. 2011. The transcription factor ABI4 is required for the ascorbic acid-dependent regulation of growth and regulation of jasmonate-dependent defense signaling pathways in Arabidopsis. Plant Cell **23**, 3319-3334.
Kka N, Rookes J, Cahill D. 2018. The influence of ascorbic acid on root growth and the root apical meristem in *Arabidopsis thaliana*. Plant Physiology and Biochemistry **129**, 323-330.
Koffler BE, Luschin-Ebengreuth N, Stabentheiner E, Muller M, Zechmann B. 2014a. Compartment specific response of antioxidants to drought stress in Arabidopsis. Plant Science **227**, 133-144.
Koffler BE, Polanschutz L, Zechmann B. 2014b. Higher sensitivity of *pad2-1* and *vtc2-1* mutants to cadmium is related to lower subcellular glutathione rather than ascorbate contents. Protoplasma **251**, 755-769.
Kotchoni SO, Larrimore KE, Mukherjee M, Kempinski CF, Barth C. 2009. Alterations in the endogenous ascorbic acid content affect flowering time in Arabidopsis. Plant Physiol **149**, 803-815.
Larkindale J, Hall JD, Knight MR, Vierling E. 2005. Heat stress phenotypes of Arabidopsis mutants implicate multiple signaling pathways in the acquisition of thermotolerance. Plant Physiol **138**, 882-897.
Li B, Yang YP, Yu CM, Li SM, Chen J, Liu X, Qin HJ, Wang DW. 2014. Partial suppression of L-galactono-1,4-lactone dehydrogenase causes significant reduction in leaf water loss through decreasing stomatal aperture size in Arabidopsis. Plant Growth Regulation **72**, 171-179.
Li SH, Wang J, Yu YW, Wang FR, Dong JG, Huang RF. 2016. D27E mutation of VTC1 impairs the interaction with CSN5B and enhances ascorbic acid biosynthesis and seedling growth in Arabidopsis. Plant Molecular Biology **92**, 473-482.
Lim B, Smirnoff N, Cobbett CS, Golz JF. 2016. Ascorbate-deficient *vtc2* mutants in Arabidopsis do not exhibit decreased growth. Frontiers in Plant Science **7**, 1025.
Lopez-Carbonell M, Munne-Bosch S, Alegre L. 2006. The ascorbate-deficient vtc1 Arabidopsis mutant shows altered ABA accumulation in leaves and chloroplasts. Journal of Plant Growth Regulation **25**, 137-144.

Mukherjee M, Larrimore KE, Ahmed NJ, Bedick TS, Barghouthi NT, Traw MB, Barth C. 2010. Ascorbic acid deficiency in Arabidopsis induces constitutive priming that is dependent on hydrogen peroxide, salicylic acid, and the NPR1 gene. Molecular Plant-Microbe Interactions **23**, 340-351.
Muller-Moulé P, Conklin PL, Niyogi KK. 2002. Ascorbate deficiency can limit violaxanthin de-epoxidase activity *in vivo*. Plant Physiol **128**, 970-977.
Muller-Moulé P, Golan T, Niyogi KK. 2004. Ascorbate-deficient mutants of Arabidopsis grow in high light despite chronic photooxidative stress. Plant Physiol **134**, 1163-1172.
Muller-Moulé P, Havaux M, Niyogi KK. 2003. Zeaxanthin deficiency enhances the high light sensitivity of an ascorbate-deficient mutant of Arabidopsis. Plant Physiol **133**, 748-760.
Munne-Bosch S, Alegre L. 2002. Interplay between ascorbic acid and lipophilic antioxidant defences in chloroplasts of water-stressed Arabidopsis plants. FEBS Letters **524**, 145-148.
Nam H-I, Shahzad Z, Dorone Y, Clowez S, Zhao K, Bouain N, Lay-Pruitt KS, Rhee SY, Rouached H. 2021. Interdependent iron and phosphorus availability controls photosynthesis through retrograde signaling. Nature Communications **12**, 7211.
Naydov IA, Mudrik VA, Ivanov BN. 2010. Light-induced hydrogen peroxide dynamics in protoplasts from leaves of both wild-type Arabidopsis and its mutant deficient in ascorbate biosynthesis. Doklady Biochemistry and Biophysics **432**, 137-140.
Olmos E, Kiddle G, Pellny TK, Kumar S, Foyer CH. 2006. Modulation of plant morphology, root architecture, and cell structure by low vitamin C in *Arabidopsis thaliana*. Journal of Experimental Botany **57**, 1645-1655.
Page M, Sultana N, Paszkiewicz K, Florance H, Smirnoff N. 2012. The influence of ascorbate on anthocyanin accumulation during high light acclimation in *Arabidopsis thaliana*: further evidence for redox control of anthocyanin synthesis. Plant Cell and Environment **35**, 388-404.
Pastor V, Luna E, Ton J, Cerezo M, García-Agustín P, Flors V. 2013. Fine tuning of reactive oxygen species homeostasis regulates primed immune responses in Arabidopsis. Molecular Plant-Microbe Interactions **26**, 1334-1344.
Pastori GM, Kiddle G, Antoniw J, Bernard S, Veljovic-Jovanovic S, Verrier PJ, Noctor G, Foyer CH. 2003. Leaf vitamin C contents modulate plant defense transcripts and regulate genes that control development through hormone signaling. Plant Cell **15**, 939-951.
Pavet V, Olmos E, Kiddle G, Mowla S, Kumar S, Antoniw J, Alvarez ME, Foyer CH. 2005. Ascorbic acid deficiency activates cell death and disease resistance responses in Arabidopsis. Plant Physiol **139**, 1291-1303.
Peto A, Lehotai N, Feigl G, Tugyi N, Ördög A, Gémes K, Tari I, Erdei L, Kolbert Z. 2013. Nitric oxide contributes to copper tolerance by influencing ROS metabolism in Arabidopsis. Plant Cell Reports **32**, 1913-1923.
Plumb W, Townsend AJ, Rasool B, Alomrani S, Razak N, Karpinska B, Ruban AV, Foyer CH. 2018. Ascorbate-mediated regulation of growth, photoprotection, and photoinhibition in *Arabidopsis thaliana*. Journal of Experimental Botany **69**, 2823-2835.
Podmaniczki A, Nagy V, Vidal-Meireles A, Toth D, Patai R, Kovacs L, Toth SZ. 2021. Ascorbate inactivates the oxygen-evolving complex in prolonged darkness. Physiologia Plantarum **171**, 232-245.
Qin C, Qian WQ, Wang WF, Wu Y, Yu CM, Jiang XH, Wang DW, Wu P. 2008. GDP-mannose pyrophosphorylase is a genetic determinant of ammonium sensitivity in *Arabidopsis thaliana*. Proceedings of the National Academy of Sciences of the United States of America **105**, 18308-18313.
Smirnoff N. 2000. Ascorbate biosynthesis and function in photoprotection. Philosophical Transactions of the Royal Society B-Biological Sciences **355**, 1455-1464.
Sultana N, Florance HV, Johns A, Smirnoff N. 2015. Ascorbate deficiency influences the leaf cell wall glycoproteome in *Arabidopsis thaliana*. Plant Cell and Environment **38**, 375-384.
Toth SZ, Nagy V, Puthur JT, Kovacs L, Garab G. 2011. The physiological role of ascorbate as Photosystem II electron donor: protection against photoinactivation in heat-stressed leaves. Plant Physiol **156**, 382-392.

Toth SZ, Puthur JT, Nagy V, Garab G. 2009. Experimental evidence for ascorbate-dependent electron transport in leaves with inactive oxygen-evolving complexes. Plant Physiol **149**, 1568-1578.
Veljovic-Jovanovic SD, Pignocchi C, Noctor G, Foyer CH. 2001. Low ascorbic acid in the vtc-1 mutant of arabidopsis is associated with decreased growth and intracellular redistribution of the antioxidant system. Plant Physiol **127**, 426-435.
Wang LY, Zhang QY, Wang F, Meng X, Meng QW. 2012. Ascorbate plays a key role in alleviating low temperature-induced oxidative stress in Arabidopsis. Photosynthetica **50**, 602-612.
Wormuth D, Baier M, Kandlbinder A, Scheibe R, Hartung W, Dietz KJ. 2006. Regulation of gene expression by photosynthetic signals triggered through modified CO_2 availability. BMC Plant Biology **6**.
Yao YN, You JJ, Ou YB, Ma JB, Wu XL, Xu G. 2015. Ultraviolet-B protection of ascorbate and tocopherol in plants related with their function on the stability on carotenoid and phenylpropanoid compounds. Plant Physiology and Biochemistry **90**, 23-31.
Zeng LD, Li M, Chow WS, Peng CL. 2018. Susceptibility of an ascorbate-deficient mutant of Arabidopsis to high-light stress. Photosynthetica **56**, 427-432.
Zhang L, Song HY, Li BH, Wang M, Di DW, Lin XY, Kronzucker HJ, Shi WM, Li GJ. 2021. Induction of S-nitrosoglutathione reductase protects root growth from ammonium toxicity by regulating potassium homeostasis in Arabidopsis and rice. Journal of Experimental Botany **72**, 4548-4564.
